# Natural variation in tetrapyrrole biosynthetic enzymes and their regulation modifies the maize chlorophyll mutant *Oy1-N1989*

**DOI:** 10.1093/plphys/kiaf431

**Published:** 2025-11-12

**Authors:** Amanpreet Kaur, Rajdeep S Khangura, Brian P Dilkes

**Affiliations:** Department of Biochemistry, Purdue University, West Lafayette, IN 47907, USA; Center for Plant Biology, Purdue University, West Lafayette, IN 47907, USA; Department of Biochemistry, Purdue University, West Lafayette, IN 47907, USA; Center for Plant Biology, Purdue University, West Lafayette, IN 47907, USA; Department of Biochemistry, Purdue University, West Lafayette, IN 47907, USA; Center for Plant Biology, Purdue University, West Lafayette, IN 47907, USA

## Abstract

Tetrapyrroles are macrocyclic compounds present in and required for all life on Earth. Mutants with defects in tetrapyrrole pathway enzymes can be used to uncover natural variation in this pathway and study pathway regulation. We report the effects of the *Oil yellow1* mutation, *Oy1-N1989*, a semi-dominant allele of subunit I in the Mg-chelatase enzyme with reduced chlorophyll biosynthesis in maize (*Zea mays*), on global gene expression and chlorophyll content. In *Oy1-N1989*/+ mutants, coordinate feedback regulation of the tetrapyrrole pathway was observed as transcriptional feedback regulation of genes encoding steps in the tetrapyrrole pathway. Natural variation in the wild-type allele at *oy1* modulated the severity of the impact of *Oy1-N1989/+* on gene expression. Previously identified cis-acting expression variation at *oy1* in wild-type plants affected similar transcriptional co-regulation of genes encoding steps in the tetrapyrrole pathway as observed in the RNA-seq of *Oy1-N1989*/+ mutants. This demonstrated that the coordinate regulation of the pathway also occurs during physiologically relevant variation in OY1 abundance. Cis variants at 7 tetrapyrrole pathway genes were linked to variation in chlorophyll accumulation in *Oy1-N1989/+* mutant or wild-type plants. Analysis of trans-acting transcriptional variation by eGWAS detected multiple transcriptional hotspots, which affected the expression of a subset of tetrapyrrole pathway genes, indicating that these genes are repeated targets of transcriptional regulation. The hotspots were encoded at locations with no known regulators of the tetrapyrrole pathway, indicating as yet undiscovered molecular mechanisms of feedback regulation operating in natural populations. The trans-regulatory hotspots coordinately regulate this pathway and may work to limit the accumulation of phototoxic intermediates.

## Teaser

Natural variation affects transcriptional feedback in the tetrapyrrole pathway in plants.

## Introduction

**Figure 1.**
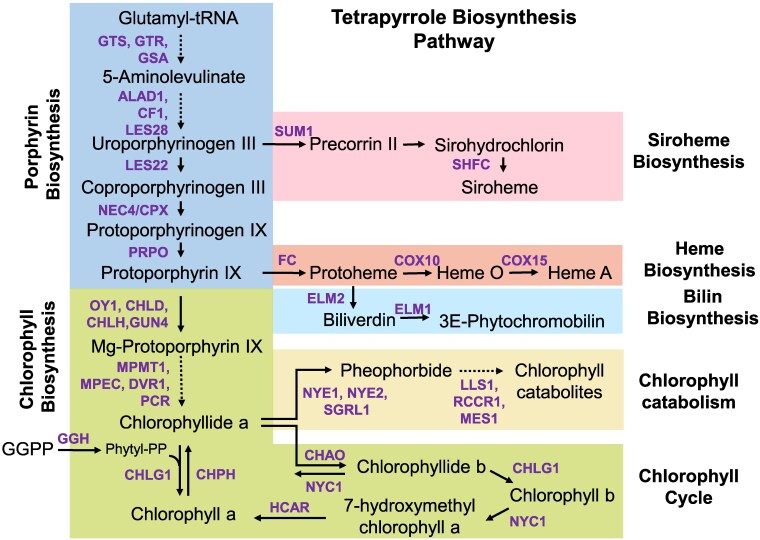
Schematic of tetrapyrrole biosynthetic pathway in maize. The enzymes catalyzing the steps in the pathway are depicted in purple. The details of abbreviations are in Supplementary Table S2.

Tetrapyrroles are macrocyclic compounds, pigments of life, involved in various biological processes fundamental to all life on Earth (Battersby 2000; Bryant et al. 2020). The diverse roles of tetrapyrroles are defined by the oxidation state of the macrocycle, the composition of sidechains, and their ability to chelate metals, including magnesium, iron, cobalt, or nickel. Tetrapyrroles in plants include chlorophyll, heme, siroheme, and phytochromobilin. All these compounds are synthesized from glutamyl-tRNA in a multi-branched pathway (Fig. 1).

Biosynthesis of 5-aminolevulinate (ALA) marks the first committed step in the biosynthesis of tetrapyrroles. In plants, ALA is synthesized from glutamyl-tRNA by 3 enzymes: glutamyl-tRNA synthetase (GTS), glutamyl-tRNA reductase (GTR), and glutamate-1-semialdehyde 2,1-aminomutase (GSA). ALA is converted to uroporphyrinogen III by the action of 3 enzymes: ALA dehydratase (ALAD), porphobilinogen deaminase, and uroporphyrinogen-III synthase. These 3 steps are conserved in all living organisms, produce phototoxic intermediates, and are common to the biosynthesis of all tetrapyrroles (Dailey et al. 2017). Little is known about their regulation in plants. The tetrapyrrole pathway bifurcates at uroporphyrinogen-III into either siroheme biosynthesis or continues to protoporphyrin IX. Protoporphyrin IX is a common precursor for the chlorophyll and heme/bilin branches. The insertion of Fe^2+^ by ferrochelatase (FC) is the first committed step for heme biosynthesis, and the insertion of Mg^2+^ to protoporphyrin IX by multimeric Mg-chelatase is the first committed step for chlorophyll biosynthesis. Mg-chelatase (CHL) consists of 3 subunits, CHLI, CHLD, and CHLH, and requires the hydrolysis of ATP (Walker and Willows 1997; Willows and Hansson 2003; Hansson et al. 2013).

Balancing the demands between branches of this pathway requires complex interplay and regulation. One aspect of this regulation is the coordinate transcriptional repression or activation of tetrapyrrole biosynthetic genes. Light signaling, circadian rhythm, phytohormones, and environmental (biotic and abiotic) stresses mediate transcriptional regulation in the tetrapyrrole biosynthetic pathway (Brzezowski et al. 2015; Kobayashi and Masuda 2016). Several transcription factors that coordinately affect the expression of genes encoding steps in tetrapyrrole metabolism are described in Arabidopsis (*Arabidopsis thaliana*) (Tanaka et al. 2011; Kobayashi and Masuda 2016). Studies of transcriptional regulation of the pathway have focused on glutamyl-tRNA reductase (GTR), encoded by *HEMA1* in Arabidopsis, as it controls the biosynthesis of ALA that serves as the precursor to all the branches of the pathway (Kobayashi and Masuda 2016). GTR activity is also regulated posttranslationally through feedback inhibition by heme and interaction with a regulatory protein FLUORESCENT (FLU), which mediates the repression of ALA synthesis under fluctuating light conditions (Tanaka et al. 2011; Wang et al. 2022). Genes encoding steps in the chlorophyll branch, including the *GENOMES UNCOUPLED4* (*GUN4*) gene and the genes encoding the subunit H of Mg-chelatase (CHLH), Mg-protoporphyrin IX monomethyl ester cyclase (MPEC), and protochlorophyllide oxidoreductases, are all known targets of transcriptional regulation (Tanaka et al. 2011; Kobayashi and Masuda 2016). The bilin pathway synthesizes phytochrome chromophore phytochromobilin, a known regulator of the tetrapyrrole pathway (Ryberg and Terry 2002; Cornah et al. 2003). Heme is metabolized by heme oxygenase, encoded by the maize (*Zea mays*) *elongated mesocotyl2* (*elm2*) gene, to synthesize phytochromobilin (Shi et al. 2013) making the pathway both light-sensitive and light-regulatory (Fig. 1).

The activities of Mg-chelatase and ferrochelatase are responsive to light signaling and concentrations of ATP. Mg-chelatase activity requires ATP, whereas ferrochelatase is inhibited by ATP (Cornah et al. 2002). Additional regulation of the chlorophyll branch of the pathway is affected by protein complex subunits. The GUN4 protein stimulates the activity of Mg-chelatase and acts as a regulatory subunit (Larkin et al. 2003). The YCF54 protein is required for Mg-protoporphyrin IX monomethylester cyclase assembly and activity but does not appear to be required in stoichiometric amounts during catalysis (Albus et al. 2012; Bollivar et al. 2014; Herbst et al. 2018; Chen and Hunter 2020; Stuart et al. 2020).

Tetrapyrroles absorb photons and can convert light energy into chemical energy to the benefit and detriment of living things. While chlorophylls and bilin contribute to photosynthesis and energy production, some intermediates produced during the synthesis and breakdown of tetrapyrroles are photoreactive. These phototoxic intermediates can be excited by light to produce free radicals and reactive oxygen species that damage cells (Busch and Montgomery 2015). To avoid ROS accumulation, biosynthesis of the phototoxic intermediates in the tetrapyrrole pathway must be tightly controlled.

Mutants with defects at various points in the pathway can help us understand the functioning of the tetrapyrrole pathway and its regulation. Several mutants defective in tetrapyrrole metabolism have been identified and characterized in maize. Weak *Mutator*-induced maize *camouflage1* (*cf1*) mutants have decreased porphobilinogen deaminase activity and develop yellow non-clonal sectors on the leaves grown in diurnal light cycles but not on leaves grown in continuous light (Huang et al. 2009). A loss-of-function allele in the same gene encodes the *necrotic3 (nec3)* mutant with necrotic bands on leaves (Huang 2009). Semi-dominant alleles of *lesion22* (*les22*) encoded by one of the uroporphyrinogen decarboxylase paralogs of maize cause the light-dependent formation of necrotic lesions on the leaves due to accumulation of phototoxic uroporphyrinogen III (Hu et al. 1998). A defect in coproporphyrinogen III oxidase in a recessive allele of *necrotic4*, *nec-t* leads to necrotic spots and yellow–green leaves (Zhao et al. 2022). These mutants have reduced chlorophyll contents and reduced accumulation of key intermediates of chlorophyll biosynthesis, including protoporphyrin IX, Mg protoporphyrin IX, and protochlorophyllide. Another mutant, *pale-green leaf (pgl)*, containing missense mutation in a gene encoding magnesium-protoporphyrin IX monomethyl ester cyclase (MPEC) led to a chlorophyll-deficient phenotype in the leaves throughout the lifespan of the plant (Xue et al. 2022). Multiple semi-dominant *Oil yellow1 (oy1)* mutants encode dominant-negative alleles of the subunit I of the Mg-chelatase enzyme and exhibit a pale green leaf phenotype with low chlorophyll accumulation when heterozygous (Sawers et al. 2006). As homozygotes, these mutants are severely deficient in chlorophyll and exhibit seedling lethality in maize (Sawers et al. 2006).

We previously demonstrated that combining natural and induced genetic variants can identify previously unknown regulation in this pathway (Khangura et al. 2019, 2020a, 2020b, 2022). The semi-dominant allele, *Oy1-N1989*, results from a missense mutation in the Mg-chelatase subunit I (Sawers et al. 2006). This encodes a dominant-negative subunit I protein that poisons the Mg-chelatase complex, which is comprised of I and D subunits arranged in a 2-layered trimer of dimers (Lundqvist et al. 2013). The third subunit of Mg-chelatase, subunit H, binds to protoporphyrin IX. The same missense mutant has been described in multiple species where it results in a defective Mg-chelatase that decouples ATP hydrolysis from Mg^2+^ chelation in the protoporphyrin IX ring (Hansson et al. 2002; Lundqvist et al. 2013). Using the *Oy1-N1989* mutant, we identified cryptic natural variation in the chlorophyll biosynthetic pathway that affected variation in mutant phenotypic severity (Khangura et al. 2019, 2020a, 2020b, 2022). Cis-regulatory natural variation at the *oy1* locus modified the chlorophyll contents of *Oy1-N1989/+* mutants (Khangura et al. 2019). In line crosses, the phenotypic consequences of *Oy1-N1989*/+ heterozygotes were increased in severity by the *oy1*^Mo17^ allele, which accumulated less OY1 transcript, and were mild with the *oy1*^B73^ allele, which accumulated more OY1 transcript. The severity of the mutant phenotype in *Oy1-N1989*/*oy1*^Mo17^ was evident in the greater reduction in chlorophyll content, lower photosynthetic rate, decreased carbohydrate accumulation, reduced stalk width, and delayed reproductive maturity as compared to *Oy1-N1989*/*oy1*^B73^ (Khangura et al. 2019, 2020a). In a GWAS study, natural polymorphisms affecting cis-regulatory expression variation at *oy1* also encoded a strong modifier of *Oy1-N1989/+* mutant severity (Khangura et al. 2019). The connection between expression GWAS (eGWAS) and phenotypic trait variation and the ability of the natural variants at *oy1* to modify the phenotype of the *Oy1-N1989* mutant led us to explore the global gene expression consequences of this mutant and the effects of natural variation on the entire tetrapyrrole pathway.

In this study, we explored transcriptional regulation of the tetrapyrrole pathway in maize using the *Oy1-N1989* mutant and its modifiers. Differential gene expression analysis in mutants displayed coordinate regulation of the tetrapyrrole pathway and compensatory effects at multiple genes encoding enzymes in tetrapyrrole biosynthesis. We performed a pathway-level exploration via eGWAS. Natural variation at *oy1* acted in trans on the accumulation of transcripts encoding multiple tetrapyrrole biosynthetic enzymes in wild-type (WT) plants. This demonstrates that even without discernible morphological effects in WT plants, regulatory variants at the natural alleles are consequential and trigger detectable transcriptional feedback regulation. The eGWAS also detected many transcriptional hotspots. Decomposition of those hotspots to single SNPs allowed us to demonstrate that natural alleles coordinately regulate the tetrapyrrole pathway, some genes in the tetrapyrrole pathway are frequent targets for transcription regulation, and development alters the pattern of transcript co-regulation. These patterns suggest an explanation for the banding patterns visible on the *cf1* mutant of maize, encoded by weak alleles of porphobilinogen synthase.

## Results

### Gene expression consequences of *Oy1-N1989* in 2 genetic backgrounds mirror their phenotypic severity

**Figure 2.**
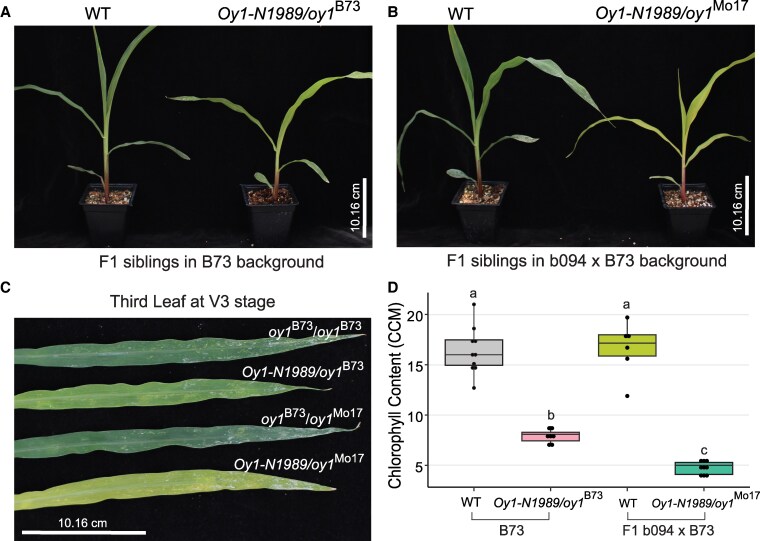
Chlorophyll pigment accumulation in seedlings of *Oy1-N1989/+* mutants is modified by the WT *oy1* allele. **A)**  *Oy1-N1989/oy1*^B73^ mutant and congenic WT (*oy1*^B73^/*oy1*^B73^) siblings in B73 background at V3 developmental stage. **B)**  *Oy1-N1989/oy1*^Mo17^ mutant and congenic WT sibling (*oy1*^B73^/*oy1*^Mo17^) in b094 × B73 hybrid background at V3 developmental stage. **C)** Third fully expanded leaves collected from *Oy1-N1989/+* mutants and their respective WT siblings. **D)** Chlorophyll contents measured from the lamina of the third fully expanded leaf at V3 developmental stage. Data are presented as boxplots, where the center lines indicates the median value and box limits represent upper (75th percentile) and lower (25th percentile) quartiles. Black dots denote the individual data points. The connecting letter report indicates statistically significant differences at *P* < 0.05 determined by Student's *t*-test.

We have previously reported changes in the chlorophyll levels, morphology, and developmental consequences of the *Oy1-N1989*/+ mutant affected by natural variation at the *vey1* (*very oil yellow1*) QTL (Khangura et al. 2019, 2020a, 2020b). The *vey1* QTL likely encodes a cis-regulatory expression polymorphism at the *oy1* locus (Khangura et al. 2019). The WT *oy1* allele derived from B73 (*oy1*^B73^) accumulates higher OY1 transcripts than the *oy1* allele derived from Mo17 (*oy1*^Mo17^). As a result, the *Oy1-N1989/oy1*^Mo17^ mutants accumulate less chlorophyll than *Oy1-N1989/oy1*^B73^ mutants (Fig. 2; Khangura et al. 2019).

**Figure 3.**
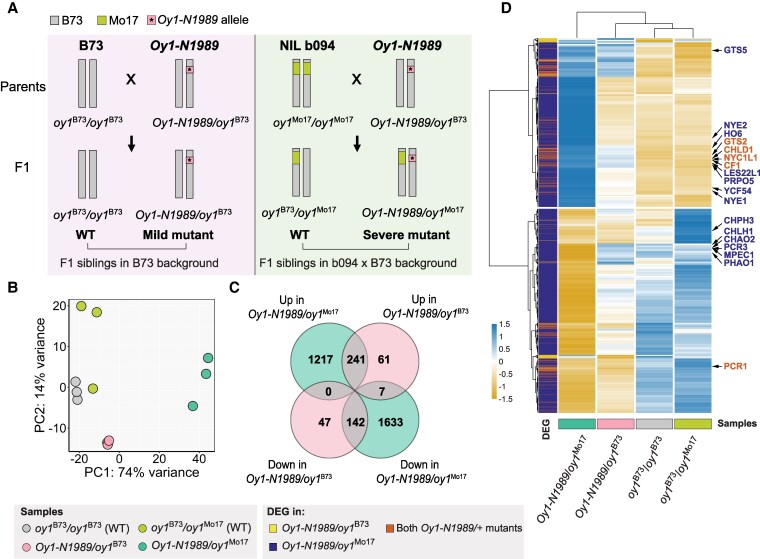
Transcriptomic analysis of *Oy1-N1989/+* mutants. **A)** The crossing scheme to generate isogenic F1 hybrids segregating 1:1 for WT plants and *Oy1-N1989/+* mutants in the B73 and b094 × B73 backgrounds. **B)** PCA of transcriptomic data. **C)** Venn diagram showing number of genes upregulated and downregulated in the phenotypically mild *Oy1-N1989/oy1*^B73^ and severe *Oy1-N1989/oy1*^Mo17^ mutants as compared to their respective congenic WT controls. **D)** Heatmap showing normalized counts of transcripts differentially accumulated in either of the 2 *Oy1-N1989/+* mutants. The transcripts highlighted in orange are differentially accumulated in both *Oy1-N1989/+* mutants and those in purple are only differentially accumulated in *Oy1-N1989/oy1*^Mo17^ mutants.

In the current study, we explored the global transcriptional consequences of *oy1*^B73^ and *oy1*^Mo17^ alleles on *Oy1-N1989* mutants in isogenic B73 genetic background. For this experiment, the inbred line B73 and a near-isogenic, line b094 that carries a homozygous *oy1*^Mo17^ introgression in the B73 background, were crossed with *Oy1-N1989/oy1*^B73^ mutants to create F1 progenies (Khangura et al. 2020b) that segregated ∼1:1 for mutant and WT siblings with alternative WT alleles at *oy1* (either *oy1*^B73^ or *oy1*^Mo17^) in a nearly isogenic B73 genetic background (Fig. 3A). The cross of B73 with *Oy1-N1989/oy1*^B73^ pollen-parent produced mildly chlorotic *Oy1-N1989/+* mutants (*Oy1-N1989/oy1*^B73^) and normal WT siblings (*oy1*^B73^*/oy1*^B73^) in the B73 genetic background. The cross of b094 with *Oy1-N1989/oy1*^B73^ pollen-parent produced severely chlorotic F1 mutant *Oy1-N1989/oy1*^Mo17^, and normal F1 WT siblings (*oy1*^B73^*/oy1*^Mo17^) in b094 × B73 hybrid genetic background (Fig. 3A). The RNA-sequencing analyses were carried out on pooled triplicates of all 4 genotypes. Principal component analysis (PCA) of the global transcriptional changes showed that the WT samples from B73 and F1 (b094 × B73) backgrounds were transcriptionally similar. In contrast, the milder phenotypic expression in *Oy1-N1989/oy1*^B73^ and more severe phenotypic expression in *Oy1-N1989/oy1*^Mo17^ mutants extended to their gene expression patterns, and these samples formed 2 distinct groups (Fig. 3B). The first principal component, PC1, captured this grouping and mirrored the loss of chlorophyll. The WT samples overlapped for PC1, a small increased value was observed for the *Oy1-N1989/oy1*^B73^ mutant samples, and a greater value was observed for all replicates of the more severely impacted *Oy1-N1989/oy1*^Mo17^ mutants.

Differential expression analysis identified 498 differentially expressed genes (DEGs) in the mildly impacted mutant, *Oy1-N1989/oy1*^B73^, as compared to WT B73 siblings (Fig. 3C). Of these 498 DEGs, transcripts of 309 were increased, and 189 were decreased in their abundance by the *Oy1-N1989* mutant. The number of DEGs was much higher in the more severe genetic background, *Oy1-N1989/oy1*^Mo17^, where mutants compared to their congenic WT siblings had a total of 3240 DEGs. Among these 3240 transcripts, 1458 were increased, and 1782 were decreased in accumulation in the mutants. A comparison of the gene expression consequences of *Oy1-N1989/+* in the severe genetic background to the effect in the mild genetic background determined that 383 (77%) of the DEGs in *Oy1-N1989/oy1*^B73^ were also differentially expressed and altered in the same direction in *Oy1-N1989/oy1*^Mo17^ (Fig. 3C). The directions of gene expression effects were substantially similar between the 2 mutant backgrounds, and multiple tetrapyrrole pathway genes clustered together across the experiment (Fig. 3D). Only 7 (1.4%) of the DEGs common between *Oy1-N1989/oy1*^B73^ and *Oy1-N1989/oy1*^Mo17^ were expressed in opposite directions, demonstrating that the gene expression changes affected in the phenotypically milder background were similarly affected when the phenotype was enhanced by the natural variants at *oy1.*

**Figure 4.**
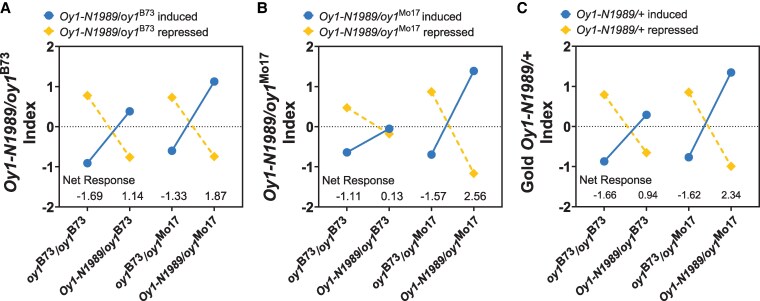
Comparison of expression effects in Oy1-N1989/+ mutants and their WT controls using expression indices. **A)** Oy1-N1989/oy1^B73^ induced and repressed indices. **B)** Oy1-N1989/oy1^Mo17^ induced and repressed indices. **C)** Gold Oy1-N1989/+ induced and repressed indices. Gold indices represent genes that were differentially expressed in both Oy1-N1989/+ mutants. The induced indices in each comparison are depicted by solid blue circle and repressed indices are depicted by a gold diamond. The index values provide relative comparison of expression effects across the 4 samples.

We investigated this further by comparing the magnitudes of expression effects between the mutants in the phenotypically milder *Oy1-N1989/oy1*^B73^ and more severe *Oy1-N1989/oy1*^Mo17^ genetic backgrounds. We calculated a Z-score from normalized transcript counts for each gene in the DEG list to measure the relative expression of the gene across the 2 mutant and 2 WT samples. The aggregate expression effects were quantified by averaging the Z-scores of all genes in the DEG list grouped by their expression direction, a value we refer to as an index (Best and Dilkes 2022; Kaur et al. 2024). This parametric comparison allows the inference of the magnitude of the effect of each mutant condition on gene expression patterns. The *Oy1-N1989/oy1*^B73^ induced index was calculated for upregulated genes and the repressed index for the downregulated genes in the mutant in this genetic background. The index for the genes induced by *Oy1-N1989/oy1*^B73^ had a higher value in the more severely affected *Oy1-N1989/oy1*^Mo17^ mutants (Fig. 4A), indicating that the *oy1*^Mo17^ allele enhanced the severity of the effects of *Oy1-N1989* on these genes. This pattern did not change when using the index values for the genes induced or repressed in *Oy1-N1989/oy1*^Mo17^ (Fig. 4B). The aggregate expression of genes, measured as an index, demonstrates that *oy1*^Mo17^ has a greater impact on expression level than *oy1*^B73^ in *Oy1-N1989/+* mutants (Fig. 4B). To ensure robustness and control bias in our gene set selection, we also calculated indices using the genes significantly affected in both mutant backgrounds. These “gold standard” DEG indices exhibit the same pattern, with greater magnitude of gene expression changes in *Oy1-N1989/oy1*^Mo17^ than in *Oy1-N1989/oy1*^B73^ (Fig. 4C). Thus, the findings of expression magnitude comport with the overall DEG numbers (Fig. 3) and mutant phenotype (Khangura et al. 2019). This global transcriptional regulation data demonstrated that *Oy1-N1989/oy1*^Mo17^ is a severe version of the *Oy1-N1989/oy1*^B73^ phenotype and is not the result of a novel or synthetic effect of the induced and natural variation brought together in these materials.

### Loss of Mg-chelatase activity in *Oy1-N1989* affects porphyrin and chlorophyll metabolism

The *Oy1-N1989/+* mutants carry a semi-dominant and dominant-negative allele of the *oy1* gene, encoding the CHLI subunit of Mg-chelatase (Sawers et al. 2006). This enzyme catalyzes the incorporation of Mg^2+^ into protoporphyrin IX, the first committed step in chlorophyll biosynthesis (Sawers et al. 2006). In our RNA-seq experiments, OY1 (Zm00001d023536) was not differentially accumulated in *Oy1-N1989/+* mutants. However, transcripts encoding Mg-chelatase subunit D (CHLD1, Zm00001d013013) increased in their accumulation in the *Oy1-N1989* mutants (Fig. 3D). The transcripts encoding CHLH1 (Zm00001d026603) were significantly reduced in *Oy1-N1989/oy1*^Mo17^ but not in *Oy1-N1989/oy1*^B73^. Other genes encoding steps in chlorophyll biosynthesis and breakdown were also affected. Transcripts of *protochlorophyllide reductase1* (*pcr1;* Zm00001d001820) were decreased in both mutants. In addition, transcripts of *Mg-protoporphyrin ester cyclase 1* (*mpec1*, Zm00001d008230), *pcr3* (Zm00001d013937), and homologs of chlorophyllide an oxygenase, *chao2* and *phao1* (Zm00001d011819 and Zm00001d042026), were decreased in the *Oy1-N1989/oy1*^Mo17^ mutant. Thus, except for the CHLD1 transcript, DEGs in the chlorophyll biosynthetic pathway were reduced in *Oy1-N1989*/+ mutants. The transcripts of genes in the porphyrin pathway leading to the formation of protoporphyrin IX showed an increased accumulation in *Oy1-N1989/+* mutants (Fig. 3D). Transcripts of *glutamate tRNA synthetase* (*gts2*, Zm00001d015037) and *camouflage1* (*cf1*, Zm00001d015366), which encodes a hydroxymethylbilane synthase, were increased in both the *Oy1-N1989/oy1*^B73^ and *Oy1-N1989/oy1*^Mo17^ mutants. In addition, mutants enhanced by the *oy1*^Mo17^ allele also accumulated transcripts of *glutamyl-tRNA synthetase5* (*gts5*, Zm00001d048372), *lesions22-like1* (*les22l1*, Zm00001d011386) which encodes an uroporphyrinogen decarboxylase, and *protoporphyrinogen IX oxidase5* (*prpo5*, Zm00001d030962). Thus, defects in Mg-chelatase subunit I in *Oy1-N1989/+* mutants caused a decrease in the accumulation of transcripts encoding chlorophyll biosynthetic genes and increased the expression of the biosynthetic genes encoding the steps prior to the formation of protoporphyrin IX.

Gene expression patterns were less consistent at transcripts encoding steps in other branches of the tetrapyrrole pathway. Transcripts of the chlorophyll degrading enzyme CHLOROPHYLLASE3 (CHPH3/CHAO1, Zm00001d031934) decreased in *Oy1-N1989/oy1*^Mo17^ (Fig. 3D). However, transcripts of other genes involved in chlorophyll catabolism, including *non-yellow coloring1-like1* (*nyc1l1,* Zm00001d013651), *non-yellowing1* (*nye1*, Zm00001d021288), and *non-yellowing2* (*nye2*, Zm00001d006211) were DEG with increased expression in *Oy1-N1989/oy1*^Mo17^ as compared to the WT. Of these, only *nyc1l1* was DEG in *Oy1-N1989/oy1*^B73^ and showed increased accumulation. The genes in other branches of the tetrapyrrole pathway, including the siroheme, heme, and bilin biosynthesis, were not differentially expressed in *Oy1-N1989/*+ mutants in either genetic background (Supplementary Table S1).

**Figure 5.**
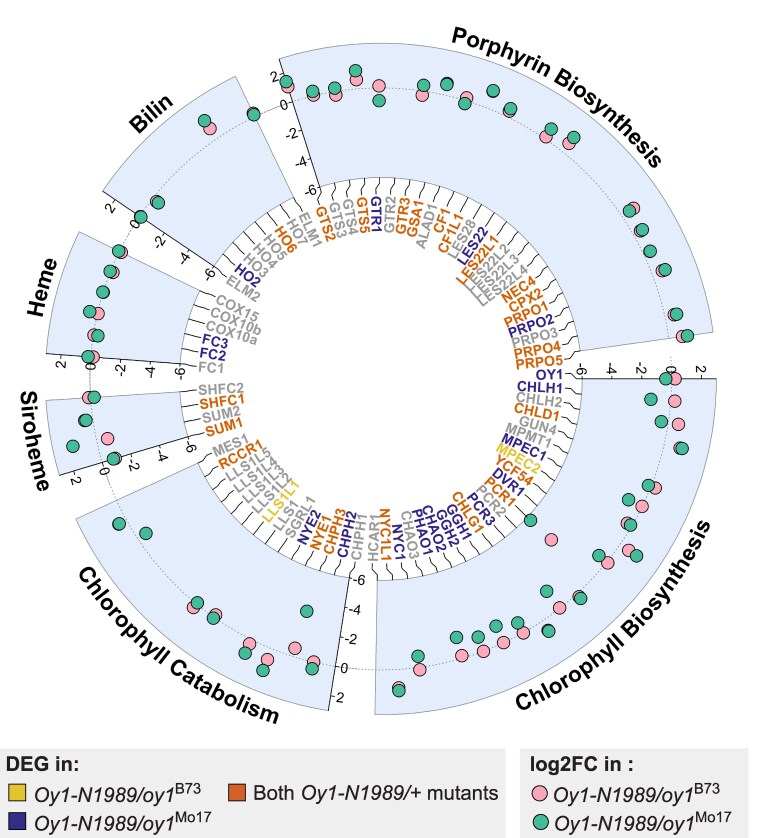
Effect of Oy1-N1989 on expression of genes encoding steps in tetrapyrrole biosynthetic pathway. Circle plot shows log2 fold change of transcripts of genes encoding steps in tetrapyrrole biosynthetic pathway in the phenotypically mild Oy1-N1989/oy1^B73^ (pink circles) and severe Oy1-N1989/oy1^Mo17^ (green circles) mutants as compared to their respective WT controls. The transcripts highlighted in orange were differentially expressed in Oy1-N1989/+ mutants in both genetic backgrounds at unadjusted *P*-value (*P* ≤ 0.05). Transcripts highlighted in yellow and purple were only differentially expressed in Oy1-N1989/oy1^B73^ and Oy1-N1989/oy1^Mo17^, respectively. Transcripts in gray were either not differentially expressed at unadjusted *P*-value cutoff of 0.05 or were not detected in our RNA-seq data.

To obtain a comprehensive view of the effect of *Oy1-N1989* on tetrapyrrole biosynthesis, we examined the expression differences at the maize homologs of all genes involved in porphyrin, chlorophyll, siroheme, heme, and bilin biosynthesis and chlorophyll degradation (Fig. 5; Supplementary Table S2). For this purpose, a *P* ≤ 0.05 was used to identify DEGs, and the direction of the effect on expression was assessed. Of the 18 expressed genes encoding steps prior to protoporphyrin IX biosynthesis, 12 were differentially expressed in *Oy1-N1989/+* mutants in both genetic backgrounds. Mutants had a greater accumulation of 11 out of these 12 differentially expressed transcripts (Fig. 5; Supplementary Table S2). In addition, transcripts of *lesion22* (*les22*, Zm00001d029074) and *protoporphyrinogen IX oxidase2* (*prpo2*, Zm00001d003214) were differentially accumulated in *Oy1-N1989/oy1*^Mo17^ mutant. These results demonstrate the existence of a transcriptional feedback mechanism that triggers the accumulation of transcripts of porphyrin biosynthetic enzymes in response to an Mg-chelatase mutant and its modulation by natural variation.

Similarly, the transcripts of 15 out of 19 expressed genes in the chlorophyll biosynthesis pathway were differentially expressed at *P* ≤ 0.05 in the severe *Oy1-N1989/oy1*^Mo17^ mutant, and only 6 were differentially expressed in the mild *Oy1-N1989/oy1*^B73^ mutant. The *Oy1-N1989/oy1*^Mo17^ mutant showed a reduced accumulation of 11 of these 15 differentially expressed transcripts for chlorophyll biosynthetic genes. Five of these 11 transcripts were also repressed in *Oy1-N1989/oy1*^B73^, but 4 were increased in their accumulation (Fig. 5; Supplementary Table S2). This demonstrates a case of epistasis where the allele at the WT copy of *oy1* altered the effect direction of transcript abundance in the *Oy1-N1989/+* mutants. What mechanisms and transcription factors mediate this feedback is unknown, let alone how they could result in such a nonlinear effect. A similar nonlinear and opposing effect of the allelic interactions between *oy1* natural variants and *Oy1-N1989* was previously observed for plant height, where the suppressing *oy1*^B73^ allele resulted in taller mutant plants compared to isogenic WT siblings (Khangura et al. 2020a). In contrast, the enhancing *oy1*^Mo17^ allele resulted in shorter mutant plants than isogenic WT siblings. It appears that the more severe loss of Mg-chelatase affected by the combination of *Oy1-N1989* and *oy1*^Mo17^ triggers reduced expression of chlorophyll biosynthetic enzymes and this feedback may further contribute to the severe loss of chlorophyll in *Oy1-N1989/oy1*^Mo17^ mutants.

Transcriptional regulators of chloroplast development and the tetrapyrrole pathway were also differentially expressed in *Oy1-N1989/oy1*^Mo17^ mutants. Transcripts of *golden plant2* (*g2*, Zm00001d039260) and *phytochrome-interacting factor4* (*pif4*, Zm00001d013130) were decreased in *Oy1-N1989/oy1*^Mo17^ as compared to its WT siblings. Transcripts of *long hypocotyl5* (*hy5*, Zm00001d015743), *phytochrome1* (Zm00001d033799), and *phytochrome2* (Zm00001d013402) were increased in accumulation in the severe mutant. The negative regulators of GA-induced gene expression DELLA-transcription factors (D8, Zm00001d033680 and D9, Zm00001d013465) decreased in accumulation in *Oy1-N1989/oy1*^Mo17^. An analysis of GO enrichment for both RNA-seq experiments is described in the Supplementary data (Supplementary Text, Fig. S1, and Tables S3 and S4).

### Cis-acting regulatory polymorphisms at tetrapyrrole pathway genes impact chlorophyll content in *Oy1-N1989/+* mutants

**Figure 6.**
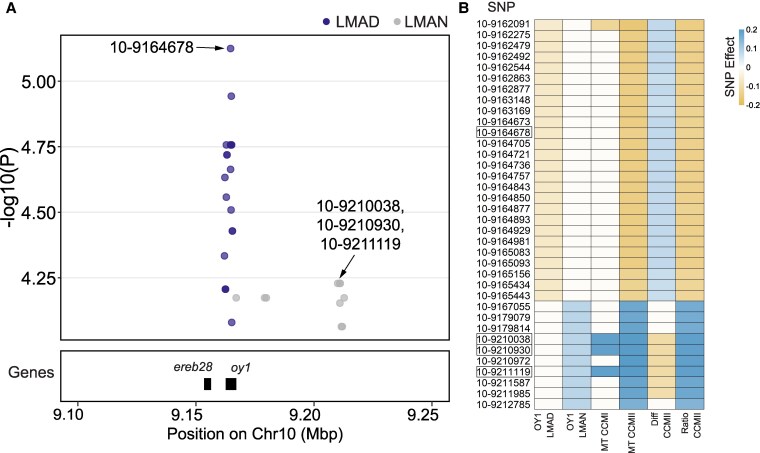
Cis-acting polymorphisms at *oy1* impact chlorophyll content in *Oy1-N1989/+* mutants. **A)** Cis polymorphisms affecting the expression of *oy1* in mature leaf tissues, LMAD (purple dots) and LMAN (gray dots), identified using eGWAS at a significance threshold of *P* ≤ 1 × 10^−4^. The top SNPs associated with OY1 transcripts in LMAN and LMAD are labeled. **B)** Effect of cis SNPs on OY1 transcripts and chlorophyll accumulation traits. Blue color indicates a positive effect of SNP on the trait and gold color indicates a negative effect on the trait value at *P* ≤ 1 × 10^−4^. White color indicates that the association between the SNP and the trait was not significant at *P* ≤ 1 × 10^−4^. For visualization, SNP effects for the traits were normalized using the respective trait means.

We performed an expression GWAS (eGWAS) for the expression of *oy1* in 3 leaf tissues (the base of the third leaf [L3Base], mature leaves collected during the day [LMAD], and mature leaves collected during the night [LMAN]) sampled from a maize diversity panel obtained from a previous study (Kremling et al. 2018). We identified cis-acting polymorphisms (within ± 50 kb of *oy1*) linked to the expression variation at *oy1* in mature leaf tissues including 26 cis SNPs in LMAD and 10 in LMAN at a *P* ≤ 1 × 10^−4^ (Fig. 6A; Supplementary Table S5). These 36 cis SNPs were evaluated for their impact on chlorophyll accumulation in *Oy1-N1989/+* mutants using data from our previous study (Khangura et al. 2019). Consistent with our previous studies (Khangura et al. 2019; Khangura et al. 2022), cis polymorphisms at *oy1* were significantly associated with chlorophyll accumulation in mutants (MT CCMI and MT CCMII), as well as mutant-to-WT chlorophyll content ratios (Ratio CCMII) and differences (Diff CCMII) for each F1 family (Fig. 6B; Supplementary Table S6). The alleles associated with reduced accumulation of OY1 were consistently associated with lower chlorophyll content in the mutant (Fig. 6B; Supplementary Table S6). No significant associations were identified for OY1 transcript abundance and WT chlorophyll contents. Thus, this exposed cryptic phenotypically impactful variation as the *Oy1-N1989* allele was required for these phenotypic effects, as expected due to previously described epistasis (Khangura et al. 2020a).

To determine if changes in the expression of other tetrapyrrole pathway genes affect chlorophyll content, we performed a similar analysis using all genes encoding steps in tetrapyrrole biosynthesis. Across 3 leaf tissues (L3Base, LMAD, LMAN), 70 of 76 tetrapyrrole pathway genes were expressed in the maize association panel (Kremling et al. 2018). An eGWAS was carried out for all 70 genes, and associations at *P* ≤ 1 × 10^−4^ were retained for further analysis. At these criteria, 57 genes, including *oy1*, had cis polymorphisms affecting gene expression variation in at least one of the 3 tissues (Supplementary Fig. S2, Table 1, and Supplementary Table S5). We intersected cis-acting SNPs affecting the accumulation of these 57 tetrapyrrole biosynthetic transcripts with the SNPs affecting variation in chlorophyll content. The greatest association overlap was observed between chlorophyll content and transcript counts from mature leaves during the day (LMAD) (Supplementary Fig. S3 and Table S6). None of these associations were as strong as the association with *oy1*. Nevertheless, cis-regulation at the Mg^2+^ chelatase subunit *chlh1* affected mutant chlorophyll content (CCMI, CCMII), and the chlorophyll contents of mutants normalized to WT siblings (difference and ratio CCM traits) (Supplementary Fig. S3 and Table S6). Increased accumulation of CHLH1 was associated with increased chlorophyll content in the mutant. Like *oy1*, the cis variation at *chlh1* did not significantly affect chlorophyll content in WT siblings. Similar relationships between transcript abundance and CCM traits were observed at 2 paralogs of *protochlorophyllide reductase* (*pcr1* and *pcr3*) that encode enzymes in the chlorophyll biosynthesis branch of the pathway (Supplementary Fig. S3 and Table S6). SNPs affecting the reduced expression of *pcr1* in LMAD, LMAN, and L3Base also reduced mutant CCMII. SNPs affecting increased expression of *pcr3* in LMAD were associated with increased mutant CCMI and ratio CCMI. Among the genes in the porphyrin pathway, only cis polymorphisms at *les22*, encoding an uroporphyrinogen decarboxylase, were significantly associated with mutant chlorophyll content. The alleles associated with increased accumulation of LES22 transcripts in LMAD and LMAN showed a reduction of chlorophyll content in mutants. A higher transcriptional activity encoding a chlorophyll precursor like *les22* is expected to increase chlorophyll content and not lower it. This inverse relationship likely indicates a feedback regulation affected by cis variation at *les22*. Cis polymorphisms linked to reduced expression of *chlorophyllase2 (chph2)*, a chlorophyll degrading enzyme, were significantly associated with higher chlorophyll accumulation in both mutant and WT plants. This inverse relationship between chlorophyll catabolism and chlorophyll content is consistent with the expectation that greater catabolism would decrease chlorophyll contents. Cis polymorphisms associated with a homolog of a heme biosynthetic gene, *ferrochelatase2* (*fc2*), were also associated with mutant chlorophyll content (CCMI, CCMII), ratio CCMII, and difference CCMII (Supplementary Fig. S3 and Table S6). The alleles linked to an increase in the expression of *fc2* negatively impacted the chlorophyll content in mutants consistent with competition between the heme and chlorophyll branches for protoporphyrin IX (Fig. 1).

**Table 1.** Summary of cis variation in transcript abundance detected by association testing of SNPs within 50 kb of the 70 genes encoding steps in the tetrapyrrole biosynthetic pathway

Tissue	Genes with cis eQTLs	Cis SNPs at *P* ≤ 1 × 10^−4^	Unique cis SNPs at *P* ≤ 1 × 10^−4^
L3Base	46	13,953	13,767
LMAD	49	11,392	11,203
LMAN	52	14,760	14,538

### Trans-regulatory natural variation repeatedly targets the same tetrapyrrole metabolism genes and results in co-regulation

**Figure 7.**
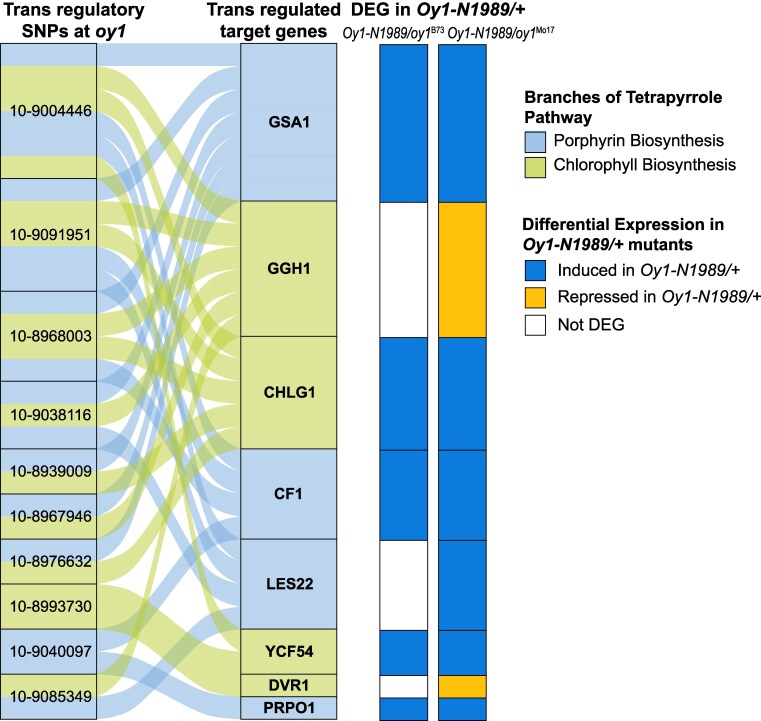
The *oy1* locus regulates the accumulation of transcripts encoded by porphyrin and chlorophyll biosynthetic genes. SNPs within ±250 kb region around *oy1* associated in trans with transcripts of at least 2 genes encoding steps in tetrapyrrole pathway in base of third leaf. Light blue and green colors indicate the branches of the tetrapyrrole pathway. The panels on the right depict the direction of effect of *Oy1-N1989/+* mutants in both genetic backgrounds on gene expression as compared to their respective congenic WTs if they exceeded an unadjusted *P* ≤ 0.05 in the RNA-seq analysis. Dark blue color indicates genes induced in the mutant, and gold color indicates genes repressed in the mutant.

We further explored natural variants affecting the expression of genes in the tetrapyrrole pathway to include trans-acting loci. SNP-transcript associations for all 70 genes in the pathway were identified through eGWAS. We identified 62 trans-acting SNPs within the *oy1* locus (±250 kb of the *oy1* gene) associated with the accumulation of 28 transcripts encoding steps in tetrapyrrole pathway in L3Base (Supplementary Table S7). Of these 62 SNPs, 10 affected the transcript abundance of at least 3 genes, consistently displaying the same effect direction for all affected genes (Fig. 7 and Table 2; Supplementary Table S7). Thus, natural variation at the *oy1* locus triggers transcriptional regulation that results in the coordinate regulation of genes encoding steps in the tetrapyrrole pathway. The 8 genes affected by these 10 SNPs encoded steps in porphyrin and chlorophyll branches of the tetrapyrrole pathway (Fig. 7). Transcript levels of 5 of these 8 genes were increased (*P* 0.05) in both *Oy1-N1989/+* mutants in our RNA-seq experiments (Fig. 7). We identified 253 SNPs at the *oy1* locus linked in trans to the expression of 25 tetrapyrrole pathway genes in LMAD (Supplementary Table S7). Of the 253 SNPs, 63 affected at least 3 genes (Supplementary Fig. S4). Similarly, 63 SNPs linked to the *oy1* locus were associated with expression variation for 17 tetrapyrrole pathway genes in LMAN. However, each of these SNPs only affected single genes. Together with the DEG analysis (Figs. 3 to 5), eGWAS strongly suggests that a transcriptional regulatory mechanism(s) affects feedback regulation in response to the loss of chlorophyll and demonstrates that this regulation is also active in WT plants. In this regard, it is worth noting that these trans-acting SNPs linked to *oy1* are more than 50 kb from the gene itself and may indicate examples of local trans effects (Albert and Kruglyak 2015). Consistent with this interpretation, each of the SNPs in Table 2 affects the accumulation of OY1 transcript in the same direction as the other tetrapyrrole genes and not in the opposite direction as would be expected for homeostatic compensation as observed in our RNA-seq experiments (Fig. 5; Supplementary Table S2).

**Table 2.** Trans-eQTLs at the *oy1* locus affecting the accumulation of 3 or more transcripts in the tetrapyrrole biosynthesis pathway

Chr-position^a^	Tissue^b^	Cis effect on OY1	eGWA OY1 *P*-value	Trans-regulated transcripts	Pathway^c^	Trans effect	Trans *P*-value
10-8968003	L3Base	−0.08	7.6E-05	GSA1	Porphyrin	−0.92	4.7E-05
				CF1	Porphyrin	−0.49	6.3E-05
				GGH1	Chlorophyll	−0.02	2.0E-05
				CHLG1	Chlorophyll	−1.27	1.9E-05
10-9004446	L3Base	−0.08	5.9E-05	GSA1	Porphyrin	−0.92	1.5E-05
				CF1	Porphyrin	−0.48	3.2E-05
				LES22	Porphyrin	−0.004	9.7E-05
				YCF54	Chlorophyll	−0.20	2.5E-05
				GGH1	Chlorophyll	−0.02	5.2E-05
				CHLG1	Chlorophyll	−1.16	3.6E-05
10-9038116	L3Base	−0.07	3.4E-04	GSA1	Porphyrin	−0.846	9.69E-05
				LES22	Porphyrin	−0.004	4.68E-05
				GGH1	Chlorophyll	−0.020	3.97E-05
10-9091951	L3Base	−0.08	5.7E-04	GSA1	Porphyrin	−0.919	6.06E-05
				CF1	Porphyrin	−0.488	6.99E-05
				LES22	Porphyrin	−0.004	9.64E-05
				GGH1	Chlorophyll	−0.022	4.15E-05
				CHLG1	Chlorophyll	−1.231	4.37E-05
10-9020908	LMAD	0.70	2.1E-04	GUN4	Chlorophyll	0.87	8.1E-05
				MPEC1	Chlorophyll	11.79	3.3E-05
				NYC1L1	Chlorophyll	0.19	5.8E-05

^a^Position in maize genome version 4.

^b^Tissue type defined in text.

^c^Branch of the tetrapyrrole pathway; see Fig. 1.

Using the eGWAS data for all 70 tetrapyrrole genes, we annotated trans-regulatory hotspots in 3 ways. First, we annotated trans-acting eQTL at the SNP level by identifying trans-acting SNPs that affected the expression of 8 or more tetrapyrrole pathway genes at a *P* ≤ 1 × 10^−4^. These were referred to as “hot SNPs.” Although the threshold for each SNP is permissive, the probability of exceeding a *P* ≤ 1 × 10^−4^ and affecting the expression of at least 8 of the 70 tetrapyrrole pathway genes is 9.8 × 10^−23^, if the associations are treated as independent tests. We tested ∼2.4 × 10^7^ SNPs on this gene set, for which a Bonferroni threshold is 2.1 × 10^−9^ for alpha < 0.05. Requiring expression effects at 8 genes for a “hot SNP” is far more stringent than a Bonferroni threshold, and every “hot SNP” surpasses this threshold by 14 orders of magnitude. Second, to increase the stringency, we filtered the eGWAS results, retaining only the top SNP (lowest *P*-value) affecting the expression of each gene in trans within a 250 kb window. Single nucleotide positions identified as top SNPs for 8 or more genes were considered “hot top SNPs.” Third, we adopted a traditional hotspot definition and annotated regions with large number of trans-acting eQTL within a genomic window to define a hotspot locus. We used 40 kb as our window size for a hotspot locus. There are 62,500 such windows in the maize genome. A 40 kb window was considered a hotspot if it contained top SNPs associated with the expression of at least 8 tetrapyrrole pathway genes. We estimated the likelihood of hotspot detection by random chance by calculating the proportion of windows that were expected to contain a SNP affecting each gene in each tissue. False discovery rates (FDRs) were estimated by permutation for each tissue.

**Figure 8.**
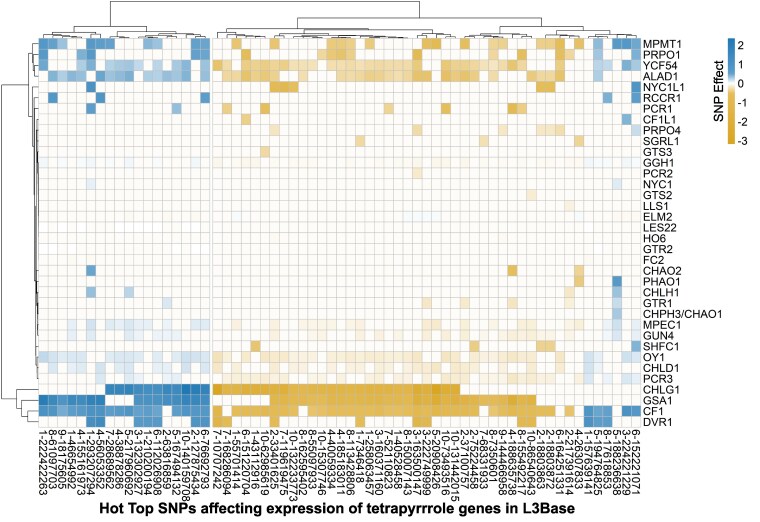
GWAS effects of hot top SNPs on the expression of associated genes in base of third leaf (L3Base). The color bar represents SNP effects. Blue color indicates a positive effect of SNP on gene expression and gold color indicates a negative effect on the expression at *P* ≤ 1 × 10^−4^. White indicates the effects were not significant at *P* ≤ 1 × 10^−4^. Dendrograms depict hierarchical clustering based on pairwise Euclidean distances calculated from SNP effect sizes. Clustering was performed using “hclust” function in R with “ward.D2” method.

We identified 408 hot SNPs in the data from L3Base, 33 in LMAD, and 27 in LMAN (Table 3; Supplementary Table S8). Candidate genes associated with each hot SNP are listed in Supplementary Table S9. Among these were 63 hot top SNPs in L3Base, 4 in LMAD, and 3 in LMAN (Table 3; Supplementary Tables S10 and S11). Candidate genes associated with the hot top SNPs are listed in Supplementary Table S12. The transcripts associated with hot top SNPs in L3Base demonstrate that some genes are more likely to be targets of trans-regulatory variation than others. For example, *cf1* was affected by the greatest number of hot top SNPs (56 out of 63), indicating that the regulation of this gene was pervasive. A similar number (47 out of 63) of hot top SNPs affected the expression level of *oy1*. As the hot top SNPs all affect multiple genes, we analyzed the allelic effect directions on all affected transcripts to explore pathway co-regulation by these natural variants (Fig. 8). Remarkably, all 63 hot top SNPs in L3Base had consistent directional effects on SNP-transcript associations with porphyrin and chlorophyll biosynthetic genes (Fig. 8; Supplementary Table S11). For instance, SNP 8-150007143 in L3Base modified the expression of 12 tetrapyrrole pathway genes (Supplementary Table S11). The allele at this position affected the expression of 11 of these genes in the same direction, including *glutamate reductase2* (*gtr2)*, *glutamate synthatase1* (*gsa1)*, *5-aminolevulinate dehydratase1 (alad1)*, *cf1*, *les22*, *chld1*, *oy1*, *ycf54*, *pcr3*, *geranylgeranyl hydrogenase1* (*ggh1)*, and *chlorophyll synthase G1* (*chlg1*), demonstrating that it alters a transcriptional regulatory pathway that coordinately regulates the chlorophyll and porphyrin branches of this pathway. The only divergent effect direction at this hot top SNP (8-150007143) was with *elm2*, which encodes heme oxygenase involved in heme catabolism and the bilin branch of the pathway. This may identify a regulatory mechanism that permits coordination between the heme and chlorophyll pathways. For example, transcriptional regulation of porphyrin and chlorophyll biosynthesis may be coordinated through a change in the abundance of the phytochrome chromophore (Inagaki et al. 2015; Kobayashi and Masuda 2019) or may result from heme oxygenase's role in retrograde signaling (Chen et al. 2024).

**Table 3.** Summary of trans-acting polymorphisms detected by expression GWAS of the 70 genes encoding steps in the tetrapyrrole biosynthesis pathway in 3 leaf tissues

Tissue	Trans-eGWAS SNPs below *P* ≤ 1 × 10^−4^	Hot SNPs^a^	Top SNPs^b^	Hot top SNPs^c^	Trans-eQTL hotspots^d^
L3Base	385,931	408	41,526	63	323
LMAD	410,722	33	45,695	4	201
LMAN	519,193	27	48,734	3	195

^a^Hot SNPs affect trans-eGWAS at *P* ≤ 1 × 10^−4^ for 8 or more genes.

^b^Top SNPs were defined within exclusive windows of 250 kb as the lowest *P*-value trans-acting SNP for each gene in each locus.

^c^Hot top SNPs encode the top SNP for 8 or more genes.

^d^Hotspots were defined as 40 kb windows encoding top SNPs that affected 8 or more transcripts.

In mature leaf collected at night (LMAN), the effect direction of all associations with 3 hot top SNPs exhibited coordinated effects on transcripts encoding porphyrin and chlorophyll biosynthetic genes. In LMAD, however, allele effects were reversed at 3 of the 4 hot top SNPs for the transcript encoded by *cf1*. For instance, the hot top SNP, SNP 10-10925524, decreased the transcript accumulation of genes encoding steps in chlorophyll biosynthesis, including *oy1*, *chld1*, *gun4*, *magnesium protoporphyrin IX methyltransferase1* (*mpmt1*), *mpec1*, *ycf54*, *divinyl reductase1* (*dvr1*), *pcr3*, and porphyrin pathway gene *alad1*, but was associated with increased transcript levels of *cf1* (Supplementary Table S11). This, and the overwhelming occupancy of *cf1* effects in the hot top SNPs in L3Base, indicates a previously unknown regulation of this pathway at the porphobilinogen synthase step. This may stop the accumulation of phototoxic intermediates, which occur immediately after the step encoded by *cf1*, by lowering the synthesis of toxic intermediates and increasing the operation of later steps to reduce the accumulation of intermediates.

A 40 kb genomic region that contained top SNPs affecting the expression of 8 or more transcripts in the tetrapyrrole pathway was annotated as a trans-eQTL hotspot. In the tissue with the most SNPs (LMAN), the average number of top SNPs per gene is 696 (48,734 top SNPs across the 70 genes; Supplementary Table S10). At this discovery rate, 0.0111 windows are expected to contain a top SNP. The likelihood of at least 8 out of 70 genes exhibiting this event is 1.18 × 10^−6^, predicting 0.07 hotspot-containing windows. However, we observed 195 trans-eQTL hotspots for LMAN (Table 3), exceeding this estimate by over 3 orders of magnitude. LMAD and LMAN have fewer top SNPs (Table 3), so we would expect even fewer hotspot-containing windows, and yet observe 201 hotspots in LMAD and 323 in L3Base (Table 3; Supplementary Table S11). To be more robust, we calculated a FDR by permutation. The number of associations per gene was tallied, randomly assigned to bins, and the number of associations per bin was determined for 1000 permutated data sets. The average number of bins with 8 or more associations was 0.092 per permutation, close to our calculated expectation of 0.07, delivering an FDR for the observed set of 195 hotspots in LMAN as 4.7 × 10^−4^. False detection rate calculated by the same procedure for the 201 observed in LMAD was 2.9 × 10^−4^ and the 323 observed in L3Base was 1.6 × 10^−4^. Thus, all approaches presented here, whether tested as alleles at the SNP level or considered as loci and tested as genomic windows, were conservatively assessed and detected far more trans-regulatory hotspots than expected by chance. This demonstrated pervasive heretofore unappreciated transcriptional co-regulation of the porphyrin pathway operating homeostatically within normal physiological ranges. Candidate genes flanking these hotspots are listed in Supplementary Table S13.

**Figure 9.**
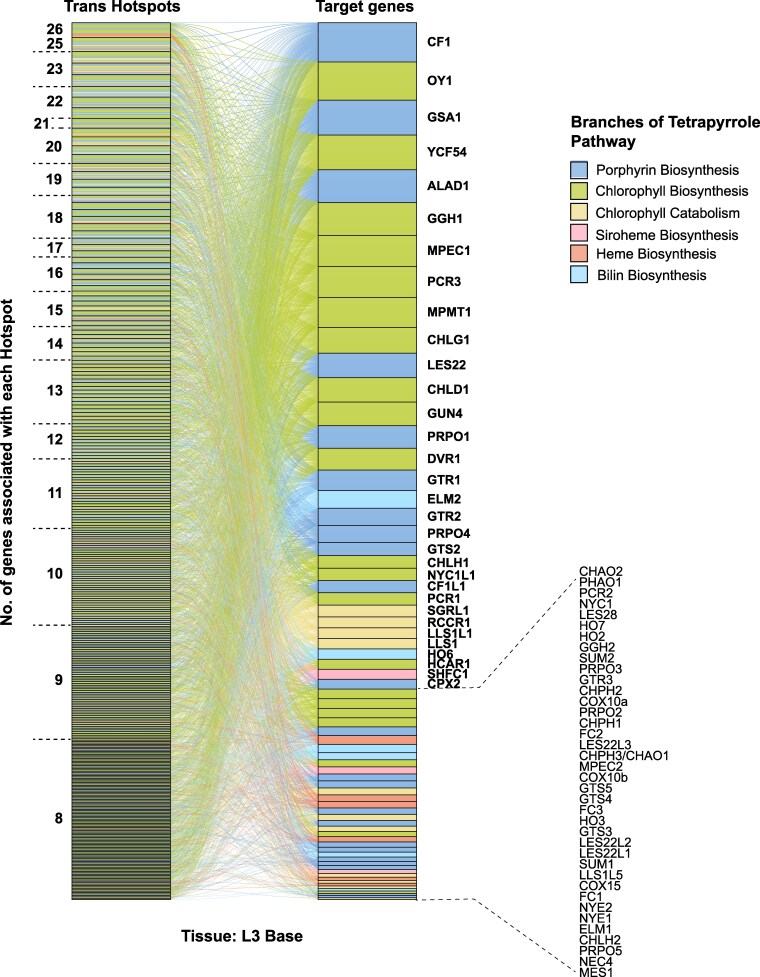
Trans-regulatory hotspots repeatedly target genes in porphyrin and chlorophyll branches of tetrapyrrole biosynthetic pathway. Trans-eQTL hotspots associated with the transcripts of 8 or more genes encoding steps in tetrapyrrole biosynthesis pathway in L3Base within a 40 kb window. The colors indicate different branches of tetrapyrrole biosynthetic pathway.

L3Base exhibited the greatest number of hotspots and a strong bias for trans-regulated genes (Fig. 9), similar to that described using the hot top SNP approach (Fig. 8; Supplementary Table S11). Trans-eQTL hotspots affected genes encoding steps in all branches of the tetrapyrrole pathway. However, porphyrin and chlorophyll branches were the most common targets of hotspot variation (Fig. 9). Similar results were observed in the mature leaf tissues (Supplementary Figs. S5 and S6). In addition, some tissue-specific effects on regulation were observed, such as the inclusion of chlorophyll catabolism genes among the repeated targets of trans-regulatory hotspots in mature leaf samples, which was consistent with the expression pattern of these genes.

The trans-eQTL hotspots accounted for a striking proportion of the overall trans-regulatory variation affecting the tetrapyrrole pathway. Eighteen genes had 10% or more of their trans-regulatory SNPs encoded within the 323 hotspots defined for the L3Base expression data (Supplementary Table S14). This represents a 30-fold enrichment of trans-regulatory SNPs within hotspots for these genes. These included transcripts from *gtr1*, *gtr2*, *gsa1*, *alad1*, *cf1*, *les22*, *protoporphyrinogen IX oxidase1* (*prpo1*), *oy1*, *chld1*, *gun4*, *mpmt1*, *mpec1*, *dvr1*, *ycf54*, *pcr3*, *ggh1*, *chlg1*, and *elm2.* Among the genes associated with trans-eQTL hotspots in LMAD, the transcripts of 3 genes, *gun4* and *mpmt1*, had 10% of their trans-regulatory SNPs within hotspots (Supplementary Table S14). In LMAN samples, the transcripts encoded by *cf1* and *mpmt1* genes had more than 9% of their trans-regulatory SNPs encoded within hotspots (Supplementary Table S14). As the plants sampled for leaf 3 and mature leaves are different biological replicates, the finding that the same genes are repeatedly affected by trans-eQTL hotspots in different tissues strongly suggests that coordinated transcriptional regulation is a feature of these pathways.

We then tested whether the divergent regulation exhibited at CF1 compared to the rest of the pathway in mature leaves sampled during the day (Supplementary Table S11) was also a feature of these hotspots. Effect direction tests are possible with individual SNPs but not with windowed hotspot loci. To test this within the hotspots, we tabulated all SNPs within the hotspots that affected the CF1 transcript and at least 2 other genes. In LMAD, 16 SNPs met these criteria, and all SNPs affected CF1 transcript accumulation in the opposite direction of other porphyrin and chlorophyll pathway genes (Supplementary Table S11). In LMAN and L3Base, 17 and 130 SNPs affected the CF1 transcript and at least 2 different genes, respectively. In LMAN and L3Base, CF1 was coordinately regulated with other pathway genes at all SNPs, indicating that at night and earlier during leaf development, the direction of co-regulation is concordant with the rest of the pathway (Supplementary Table S11). This result demonstrates a change in the transcriptional feedback mechanisms or consequences in mature leaf tissue during the day relative to the 2 others. As *cf1* encodes the last step before the production of phototoxic intermediates (Vavilin and Vermaas 2002; Huang et al. 2009; Zhao et al. 2022), this may indicate transcriptional feedback to prevent the accumulation of these metabolites during the day.

### Chlorophyll accumulation is affected by a subset of trans-eQTL hotspots affecting the tetrapyrrole pathway

We tested if the SNPs within transcriptional hotspots affected chlorophyll accumulation in the F1 association mapping experiment with *Oy1-N1989*. We tabulated all overlaps that exceeded a *P* ≤ 1 × 10^−4^ for each SNP-trait association. Given the number of SNPs within hotspots (Table 3) and 8 traits, we detected more associations than expected for each of the 3 expression sets (2 SNPs in L3Base, 5 in LMAD, and 5 in LMAN), equivalent to a false detection rate of <0.42 (Supplementary Table S15).

Single SNP tests within the hotspots were also carried out. An experiment-wide Bonferroni threshold (α ≤ 0.05) was calculated for the comparison of the SNPS within hotspots in each tissue across 8 phenotypic traits. This identified a single SNP in the L3Base hotspots and a single SNP in the LMAD hotspots that exceeded this threshold (Supplementary Table S16). In L3Base, this identified a hotspot resident SNP, 5-144452049, associated with higher chlorophyll contents of the WT F1 plants and increased the difference in CCM between WT and mutant siblings. This SNP was also associated with a lower accumulation of the ELM2 transcript. In a previous study, we demonstrated that *elm1* loss-of-function mutants synergistically enhanced the *Oy1-N1989*/+ phenotype (Khangura et al. 2022). Consistent with the synergistic interaction between *elm1* loss-of-function and *Oy1-N1989*/+ (Khangura et al. 2022), the allele at SNP 5-144452049 that decreased the abundance of ELM2 transcript decreased the CCM of *Oy1-N1989*/+ mutants (MT_CCMI: *b* = −0.71, *P* = 0.019; MT_CCMII: *b* = −1.70, *P*-value = 0.036). The opposite effect of 5-144452049 on the CCM measurements from mutant and WT siblings provides additional evidence that the heme/bilin and chlorophyll pathways engage in or respond to meaningful regulatory crosstalk affected by natural variation in maize. This SNP is located at a position with no annotated genes within 10 kb between a homolog of the Arabidopsis stomatal patterning genes *SPEECHLESS* and *MUTE* (*bhlh139*; Zm00001d016095; Mano et al. 2023) on the left, and a gene encoding a required tethering component of ferredoxin NADP + reductase (Zm00001d016100) on the right (Jurić et al. 2009). Mutations in either of these genes could potentially alter photosynthesis and induce feedback on the pathway. In LMAD, the hotspot resident SNP, 5-211181311 affected the expression of the *les22l2* gene, encoding a paralog of the uroporphyrinogen decarboxylase gene, and passed a Bonferroni threshold for effects on chlorophyll accumulation. This SNP encoded an allele that increased the accumulation of chlorophyll in the mutant F1 plants. This SNP is linked to the cytokinin receptor *hairy sheath frayed1/zmhistidine kinase1* (*hsf1*; Zm00001d017977; Muszynski et al. 2020), indicating a possible link between cytokinin signaling and tetrapyrrole pathway gene expression in maize. No SNP passed the Bonferroni threshold in the overlap with the LMAN hotspots. These data highlight that there are cases of significant overlaps, even at a conservative Bonferroni corrected *P*-value cutoff, between phenotypic impacts and the expression covariation in this pathway. However, the overwhelming majority of detected trans-regulatory hotspots did not cross this threshold for the accumulation of chlorophyll in WT plants nor adjust the phenotype in response to the limitation on chlorophyll biosynthesis affected by the *Oy1-N1989* mutant.

The trans acting SNP 5-211181311 linked to *hsf1* decreased the accumulation of LES22L2 and increased mutant CCMI and ratio CCMI traits. The role of the *les22*-like genes in maize is unknown. We explored the phylogenetics of these genes, as they were not previously investigated, and determined that they derive from duplication of uroporphyrinogen decarboxylase in the green plant lineage (Viridiplantae) before the divergence of the lineages leading to *Chlamydomonas* (*Chlamydomonas reinhardtii*) and the land plants and both lineages have retained members of both duplicates for more than a billion years (Supplementary Fig. S7). In maize and Arabidopsis, one clade contains *les22* (Zm00001d029074) of maize and *HEME2* (AT2G40490) of Arabidopsis, while the sister clade contains at least 4 paralogs in maize, *les22-like1* through *les22-like4* (Zm00001d011386, Zm00001d011387, Zm00001d044186, Zm00001d044321), and *HEME1* (AT3G14930) of Arabidopsis. The vast majority of the tetrapyrrole pathway enzymes from Arabidopsis can complement their respective yeast mutant, but notably, neither HEME1 nor HEME2 of Arabidopsis can complement the yeast mutant defective in this step (Kachroo et al. 2017). The phylogeny, inability of these genes to complement yeast, and detection of eQTL at trans-acting hot spots affecting the expression of these genes illuminate a gap in our understanding of the uroporphyrinogen decarboxylases of the green plant lineage, encoded in maize by *les22* and 4 paralogs in an ancient sister lineage shared with *Chlamydomonas*.

### Pathway-level expression variation detects OY1 as a trans-eQTL hotspot affecting the expression of chlorophyll biosynthesis genes

To further explore the coordinated transcriptional regulation of the chlorophyll branch of the pathway by natural variation in maize, we identified 22 genes as chlorophyll pathway members (Fig. 5; Supplementary Table S2). We calculated an index to estimate the aggregate effects of this pathway in each of the 3 leaf tissues. Using the chlorophyll biosynthesis index as our trait, we performed GWA to identify SNPs associated with coordinated expression changes in the chlorophyll branch of the pathway (Supplementary Fig. S8 and Table S16). Significant SNP associations at the *oy1* locus were identified in L3Base and LMAD, indicating that the polymorphism at *oy1* influenced transcript abundance in the chlorophyll biosynthesis pathway (Supplementary Fig. S8; Table 4). Three of 5 SNPs affecting the chlorophyll biosynthetic index were also trans-eQTL affecting the accumulation of transcripts encoded by *gun4*, *mpec1*, and *nyc1l1* (Table 2). Two of these SNPs (10-8968003 and 10-9004446) are shown in Fig. 7. Like the hot top SNPs and most hotspots, these SNPs did not have significant associations with mutant or WT chlorophyll accumulation.

**Table 4.** SNPs at the *oy1* locus associated with the chlorophyll biosynthesis index

Chr-position	Tissue	SNP effect	*P*-value
10-9020908	LMAD	0.154	6.36E-05
10-8959176	L3Base	−0.197	3.84E-05
10-8965572	L3Base	−0.175	4.59E-05
10-8968003	L3Base	−0.180	8.46E-05
10-9004446	L3Base	−0.168	9.21E-05

A genome-wide association using the chlorophyll biosynthesis index from the 3 tissues as traits identified 11 SNPs at *P* ≤ 1 × 10^−7^ (Table 5). Ten of these 11 SNPs were detected in our trans hotspot analysis as hot SNPs, hot top SNPs, or within the hotspots (Table 5). Only a single index hit, on chromosome 2 at 148 Mb, was neither detected as a hotspot nor a hot SNP (Table 5). This demonstrates that counting significant associations to identify hotspots/hot SNPs and the parametric sums of expression effects of the index calculation can arrive at the same loci.

**Table 5.** SNPs associated with the chlorophyll biosynthesis index at a *P* < 1 × 10^−7^

Chr-position	SNP effect	*P*-value	Trans regulatory hotspot class	Tissue
2-2949376	−0.221	2.64E-08	Hot SNP	LMAN
2-149706803	−0.220	7.43E-08		LMAD
2-183572149	−0.242	1.19E-08	Within hotspot	LMAD
7-13071884	−0.257	9.72E-08	Hot top SNP	LMAD
8-69497004	0.229	4.15E-08	Within hotspot	L3Base
8-69506048	0.238	3.73E-08	Within hotspot	L3Base
8-69509342	0.232	8.90E-08	Within hotspot	L3Base
8-69547802	0.228	7.70E-08	Within hotspot	L3Base
8-69571295	0.233	2.68E-08	Within hotspot	L3Base
9-77570763	−0.230	7.32E-08	Within hotspot	L3Base
10-10925524	−0.160	6.80E-08	Hot top SNP	LMAN

## Discussion

### The WT allele at *oy1* determines the severity of gene expression consequences in *Oy1-N1989/+* mutant

This study examined the transcriptional consequences of variation in the *oy1* gene encoding subunit I of the Mg-chelatase enzyme in maize. We used the semi-dominant *Oy1-N1989* dominant-negative mutant allele in heterozygous plants with differing phenotypic severity, conditioned by the WT allele at the *oy1* locus. Consistent with the morphological impacts of natural variation on *Oy1-N1989/+*, mutant heterozygotes carrying the WT *oy1*^Mo17^ allele showed a more severe effect on gene expression than mutants carrying the *oy1*^B73^ allele. This was evident from the number of DEGs in RNA-seq analysis, where the severely impacted mutant affected more DEGs than the phenotypically milder mutant compared to their congenic WTs (Fig. 3). Among the DEGs affected by both mutants, transcript levels of the shared genes were impacted in the same direction by *Oy1-N1989* irrespective of the WT allele at *oy1*. The severe phenotype of *Oy1-N1989/oy1*^Mo17^ resulted in a greater fold-change in the expression of DEGs due to *Oy1-N1989/+* in both backgrounds (Fig. 3). This was further demonstrated using expression indices, revealing greater changes in transcript accumulation in the severe mutants regardless of the gene sets tested (Fig. 4). These results indicate that the genetic background affected by the *oy1* locus determined the severity of gene expression effects due to *Oy1-N1989*. The use of an expression index as a tool for analyzing coordinate regulation of gene expression was validated by the fact that transcriptional hotspots colocalized with GWAS peaks obtained using index values (Table 5).

### Variation in expression of *oy1* WT alleles in diverse maize lines affects the severity of the *Oy1-N1989*/+ mutant phenotype

We previously demonstrated that cis-regulatory polymorphism at *oy1* is associated with a greater accumulation of OY1 transcripts from the B73 allele using NILs, RILs, and F1 hybrids (Khangura et al. 2019). Lower expression of WT *oy1* increased the phenotypic severity of *Oy1-N1989/+* (Khangura et al. 2019). In this study, we compared the cis polymorphisms at the *oy1* locus affecting OY1 transcript accumulation with the SNPs affecting chlorophyll accumulation in mutant F1 hybrids. The cis-acting SNPs affecting OY1 transcript accumulation in our 2 mature leaf tissues (LMAD and LMAN) were significantly associated with mutant chlorophyll traits (Fig. 6). Two sets of cis-regulatory SNPs were separated by ∼2 kb and were encoded at opposite ends of the gene (Fig. 6). These SNPs were linked to 2 different alleles, both of which were required to describe the suppression of the *Oy1-N1989/+* phenotype and allele-specific expression by cis-acting polymorphisms (Khangura et al. 2019, 2020a, 2020b). The effect of SNPs at one end of the gene influenced OY1 accumulation in LMAD and was associated with chlorophyll accumulation at the later time point (CCMII; Fig. 6). OY1 transcript accumulation in LMAN was affected by SNPs at the opposite end of the gene that were associated with chlorophyll accumulation at both the earlier (CCMI) and later (CCMII) time points with a stronger effect at the later time point (Fig. 6), suggesting progressive accumulation and greening of leaves over time. These 2 sets of SNPs showed opposite effect directions relative to the reference (B73) genotype: non-reference SNP genotypes at the LMAD positions increased OY1 expression and chlorophyll accumulation, whereas those at LMAN positions decreased both. Cis-regulatory alleles that increased the expression of *oy1* were consistently associated with higher chlorophyll contents in the mutants.

### Loss of Mg-chelatase activity affects the expression of tetrapyrrole pathway genes

In plants, all tetrapyrroles are synthesized from a glutamyl-tRNA precursor in a multi-branched pathway (Tanaka and Tanaka 2007; Brzezowski et al. 2015; Bryant et al. 2020). In this study, we identified 70 maize genes encoding steps in all the branches of the tetrapyrrole pathway (Fig. 1; Supplementary Table S2). We detected pathway-level effects through the coordinated regulation of successive steps and branches (Fig. 5). *Oy1-N1989*/+ plants displayed increased transcript levels for genes encoding the steps in the porphyrin biosynthesis up to protoporphyrin IX (Figs. 1 and 5). This demonstrates the existence of a transcriptional regulation of the pathway that affects more than just glutamyl-tRNA reductase in response to mutation of Mg-chelatase. The altered transcript levels of upstream ALA and porphyrin biosynthetic genes suggest that a transcriptional mechanism either senses Mg-chelatase activity or responds to the accumulation of biosynthetic intermediates. The accumulation of some intermediates between ALA and protoporphyrin IX in the presence of light and oxygen damages the cell (Vavilin and Vermaas 2002). If these intermediates had accumulated in the mutant, we would expect phototoxic damage phenotypes in *Oy1-N1989/+* mutants, but none were observed. The lack of such phenotypes suggests that the increased transcription from these genes did not cause the accumulation of toxic intermediates in the mutant. The reduced transcript abundance of *sum1* (Fig. 5; Supplementary Table S2), which encodes the first committed step in the siroheme branch, suggests a compensatory regulatory mechanism may exist to reduce flux in the siroheme pathway during a loss of chlorophyll. Other genes in this branch did not show a similar decrease in expression. We did not observe transcriptional regulation of the heme branch as the transcript levels of genes encoding steps in the heme and phytochromobilin branch were not affected in *Oy1-N1989*/+ mutants.

*Oy1-N1989/+* altered the expression of genes encoding steps in the chlorophyll biosynthesis branch. In both genetic backgrounds, *Oy1-N1989/+* increased CHLD transcripts, demonstrating a compensatory feedback regulation at the transcription of Mg-chelatase. Prior work with a rice loss-of-function allele in the *OsCHLI* gene resulted in similar gene expression patterns with increased accumulation of upstream porphyrin biosynthesis steps, increased CHLD, and decreased GUN4 transcripts (Shim et al. 2023). This similarity provides further confirmation of the dominant-negative nature of the *Oy1-N1989* allele. The consequences of *Oy1-N1989* and its modifiers on the transcript levels of genes encoding other enzymes were more complicated. When the *oy1^B73^* allele was present, transcript levels of CHLH, a subunit of Mg-chelatase, showed a compensatory increase. But when the *oy1^Mo17^* allele was present, transcript levels of CHLH were decreased, raising the possibility that *oy1^Mo17^* enhanced the phenotype of *Oy1-N1989/+* mutants by reducing the accumulation of transcripts encoding steps in the chlorophyll biosynthetic pathway rather than affecting compensatory upregulation of these steps. The *oy1^Mo17^*-enhanced *Oy1-N1989/+* mutants also had significantly fewer transcripts from genes in the chlorophyll cycle (Figs. 1 and 3; Supplementary Table S1). This suggests a distinct transcriptional response to severe reductions in chlorophyll accumulation, perhaps because these steps would require less flux when less metabolite was present. This demonstrates yet another detection of a set of co-regulated genes affected by an understudied transcriptional regulation in this pathway. Similarly, transcripts of genes encoding steps in chlorophyll catabolism, including *nye1*, *nye2*, and *red chlorophyll catabolite reductase1* (*rccr1*), were all reduced in accumulation in *Oy1-N1989/+* mutants, suggesting a decrease in chlorophyll catabolism when chlorophyll was limited and demonstrating a transcriptional regulatory mechanism acting at the end of the pathway as well. Once again, exploring the gene expression consequences of metabolic mutants highlights regulatory pathways not previously detected.

### Natural variation affected transcriptional co-regulation of genes encoding steps in tetrapyrrole biosynthesis

One disadvantage to using mutants is the outsized impact they often have on metabolism and the potential for complex secondary effects. Death, for example, is the ultimate pleiotropic phenotype. By contrast, metabolic feedback in biochemical pathways is constructed during evolution in response to a narrower physiological range of gene products (transcripts and protein) and metabolite levels. One way to avoid the pitfalls of overinterpreting mutant phenotypes and to seek physiologically relevant regulation is to explore natural variation. In this study, eGWAS of 70 genes in the tetrapyrrole pathway in WT maize lines identified abundant cis and trans regulation affecting these genes in 3 leaf tissues. Remarkably, SNPs linked to the *oy1* locus were identified as trans-regulators of the pathway. This included SNPs affecting the expression of 25 genes in LMAD, 17 in LMAN, and 28 in L3Base at the permissive 1 × 10^−4^  *P*-value cutoff (Supplementary Table S7). This suggests that the feedback affected by the *Oy1-N1989* allele is not an artifact of an unusual mutant allele and can also be affected by natural variation at this locus. However, it remains formally possible that another linked gene affects transcriptional regulation of the tetrapyrrole pathway. It demonstrates that these genes are co-regulated and that the natural variants are of sufficient consequence to cause compensatory transcriptional regulation of genes encoding steps in the tetrapyrrole pathway. This was further demonstrated through GWAS using gene expression index value derived from 22 genes in the chlorophyll biosynthesis and chlorophyll cycle. This aggregate trait, a chlorophyll biosynthesis index, was also affected by SNPs linked to *oy1* (Table 4). Together with the RNA-seq data from *Oy1-N1989/+* mutants in the 2 genetic backgrounds, these data indicate that variation in *oy1* triggers a transcriptional checkpoint regulating the tetrapyrrole biosynthetic pathway.

To further explore the transcriptional regulation of the tetrapyrrole biosynthetic pathway, we identified hot top SNPs that affected the expression of 8 or more genes in trans. The analysis of the genes affected by 63 hot top SNPs showed that transcript levels of nearly all genes affected by these trans-regulatory hotspot alleles were co-regulated (Fig. 8). These genes encode the initial common steps of the tetrapyrrole pathway and steps in the chlorophyll branch. Like the coordinate direction of expression effects (Fig. 8), a subset of steps in the porphyrin and chlorophyll pathway were more commonly affected by transcriptional hotspots than other steps of the pathway when assessed either as windowed hotspot loci or as single hot top SNPs (Figs. 8 and 9).

Only one gene displayed divergent trans-regulatory consequences in the hot top SNPs: *cf1*. This gene is only divergently affected in expression level in mature leaves during the day. It may be that the different regulation in the leaf base and the mature leaf underlies the unusual phenotype expression. The SNPs associated with trans hotspots in LMAD had the opposite effect on the expression of *cf1* compared to other co-regulated porphyrin and chlorophyll biosynthetic genes in the pathway. In contrast, transcript levels of *cf1* were coordinately regulated with transcripts of porphyrin and chlorophyll biosynthetic genes in LMAN and L3Base. The gene *cf1* encodes a porphobilinogen deaminase that catalyzes the conversion of porphobilinogen to hydroxymethylbilane, the precursor of uroporphyrinogen III. Hydroxymethylbilane is unstable and can spontaneously cyclize to form photoreactive uroporphyrinogen I. The *cf1* gene might be differently regulated in mature leaf tissues to prevent the accumulation of phototoxic intermediates and thus prevent photodamage. One could argue that if there is a negative regulation of *cf1* during the day to reduce the accumulation of phototoxic intermediates, we should see similar effects in the base of third leaf, which was also collected during the day. However, we do not see opposing regulation of *cf1* in this tissue. This suggests that regulation of the pathway is dependent on the developmental stage. Perhaps consonant with this, both the weak alleles found in *cf1* mutants and the strong loss-of-function allele encoded by *necrotic3* result in diurnal bands of brown tissue in seedlings when grown in light:dark diurnal cycles (Huang 2009; Huang et al. 2009). The upregulation of enzymes upstream in porphyrin metabolism by *Oy1-N1989* (Fig. 5; Supplementary Table S2) and feedback regulation of *cf1* by alleles of *oy1* (Supplementary Table S7) might explain the suppression of *cf1-m2* by *Oy1-N700*. The *cf1-m2* allele is encoded by a mu insertion in the 5′UTR limiting transcription (Huang et al. 2009) as are all 4 camouflage alleles. In this light, the fact that *oy1* in WT maize acted as a trans regulator of *cf1* and that *cf1* showed changes in expression in *Oy1-N1989/+* mutants may explain the paradoxical suppression of the mutant phenotype in *cf1* by the mutation of the downstream *Oy1-N700/+*.

### Natural variation in the expression of tetrapyrrole pathway genes impacts the severity of *Oy1-N1989* phenotype

Investigation of natural variation affecting the expression of tetrapyrrole metabolism genes identified cis-regulatory variation linked to these genes (Supplementary Fig. S3). As described above, SNPs affecting cis-acting regulation of the *oy1* locus in mature leaf tissues also influenced the chlorophyll contents of the mutants in F1 association mapping GWAS experiments (Fig. 6). All cis variants associated with *oy1* expression were also associated with variation in mutant chlorophyll contents, but not WT chlorophyll contents from our previous F1 association mapping experiments (Fig. 6) (Khangura et al. 2019). The joint performance of these SNPs across 2 different experiments represents true biological replicates and even utilizes inbreds and F1 hybrids as the basis of the material. We explored whether SNPs affecting cis-regulatory expression variation at other tetrapyrrole pathway genes also affected the mutant chlorophyll content in the F1-association mapping experiment. By cross-tabulating the impact of the cis variants at all genes on chlorophyll content in both the WT and *Oy1-N1989* mutant F1 hybrids, we identified additional natural variants impacting both the gene expression and chlorophyll content (Supplementary Fig. S3 and Table S6). However, unlike the results obtained for *oy1*, not all cis variants associated with the expression of other tetrapyrrole pathway genes showed an impact on chlorophyll content even at a suggestive *P*-value cutoff of 1 × 10^−4^.

### Transcriptional hotspot analyses rely on a false and dangerous assumption

All assessments of hotspots, here and elsewhere, use a null hypothesis that relies on an assumption that gene expression is independent in each sample. While convenient and necessary for these calculations, this is naive and false and will be trivially rejected in any experiment where the sample size is large enough. The tens of thousands of gene expression estimates from each sample share an abiotic environment, sampling time, developmental stage, wounding history, and biotic influence and so covariance in gene expression will be greater within a sample than between neighbors. An assumption of independence short-hands all causes of expression correspondence, and any fortuitous linkage with SNP variation, as genetic causation. As a result, we expect hotspot calculations to overestimate the number of hotspots by whatever degree of inflation is caused by experimental design flaws and stochastic alignment of SNP variation and environmental features. Future molecular genetic tests of single-locus effects on feedback regulation are necessary to determine the mechanisms of tetrapyrrole regulation.

## Materials and methods

### Plant material

The mutant allele *Oy1-N1989* was obtained from the Maize Genetics COOP and backcrossed for 8 generations with B73, as described previously (Khangura et al. 2019). Pollen from the *Oy1-N1989/+* mutant introgressed into the B73 inbred background (*Oy1-N1989/oy1*^B73^) was crossed to B73 to generate F_1_ hybrids segregating 1:1 for mutant and WT siblings. The mutant *Oy1-N1989/oy1*^B73^ was also crossed to a near-isogenic line, b094, carrying an introgression of the Mo17 allele at *oy1* (*oy1*^Mo17^) in the B73 background (Khangura et al. 2019, 2020b). The progeny of this cross resulted in ∼1:1 segregation of mutant (*Oy1-N1989/oy1*^Mo17^) and WT plants (*oy1*^B73^*/oy1*^Mo17^) in a b094 × B73 F1 hybrid background (Khangura et al. 2020b). Plants were grown at the Purdue Agronomy Center for Research and Education (ACRE) in West Lafayette, Indiana, during the summer of 2017. Standard fertilization, weed suppression, and pest control practices for maize (*Z. mays*) cultivation were followed.

### Chlorophyll content measurements

The chlorophyll contents of maize seedlings were measured nondestructively using a Chlorophyll Content Meter (Model CCM-200 plus, Opti-Sciences, Inc., Hudson, NH). Readings were taken from the leaf lamina of the third fully expanded leaf in both the WT and mutant siblings at the V3 developmental stage.

### RNA-sequencing and transcriptomic analysis

We collected the third leaf from field-grown plants at the V3 developmental stage. Six individuals of each genotype were pooled to make one biological replicate. Three biological replicates were collected from *Oy1-N1989/oy1*^B73^ mutants in B73, *Oy1-N1989/oy1*^Mo17^ mutants from b094×B73 F1 crosses, and their congenic WT siblings. Total RNA was extracted using TRIzol reagent (Invitrogen, CA, USA). The concentration and integrity of RNA were assessed using a NanoDrop 2000c spectrophotometer (Thermo Scientific, Waltham, MA). Novogene (Sacramento, CA) performed library construction and sequencing using the NovaSeq 6000 sequencing platform (Illumina, San Diego, CA).

Sequenced reads were aligned to the maize reference (B73 RefGen_v4) genome (Jiao et al. 2017) downloaded from MaizeGDB (https://download.maizegdb.org/Genomes/B73/Zm-B73-REFERENCE-GRAMENE-4.0/). Alignment was done using *bowtie2 v2.2.8* (Langmead and Salzberg 2012) and the gene models from the maize reference genome version 4 annotation were used to identify splice junctions. The number of reads aligned to each gene was used to derive counts using *htseq-count* (Anders et al. 2015). Differential expression analysis was performed using DEseq2 v1.26.0 (Love et al. 2014). Transcripts with |log_2_ fold change| ≥ 1 and FDR adjusted *P* ≤ 0.05 were considered as differentially expressed (Supplementary Table S1). Unfiltered DESeq2 outputs are in Supplementary Table S17. Heatmaps were generated using the *pheatmap* package in R (Kolde 2019).

To calculate the index value for *Oy1-N1989/+* mutants and their congenic WTs, we derived Z-scores for the accumulation of each gene in the DEG sets. The Z-score of each gene per sample was calculated from the normalized transcript counts as follows:


$$Z\text{-}\mathrm{scor}e_{\mathrm{geneA}}=\;\frac{Normalized\;counts\;of\;gene\;A\;-Average\;counts\;of\;WT\;and\;mutant}{Standard\;Deviation}$$

The index value for each sample was obtained by averaging the Z-scores for a set of genes identified as differentially expressed.

Gene Ontology (GO) enrichment analysis for DEGs was performed using a web-based server *AGRIGO v2.0* (Tian et al. 2017) using the following parameters: Fisher's test, with Yekutieli (FDR under dependence) multi-test adjustment (Benjamini and Yekutieli 2001). Plant GO Slim was selected as the GO type. GO terms with FDR < 0.05 were selected as significant. Data were visualized using the *GOplot* package in R (Walter et al. 2015). Z-scores for each GO term in the GOplot were calculated as follows:


$$Z\text{-}\mathrm{scor}e_{\mathrm{GO}\;\mathrm{term}}=\;\frac{No.\;of\;upregulate\;genes\;-No.\;of\;downregulated\;genes}{\sqrt{upregulated\;+\;downregulated}}$$

### Identification of genes involved in tetrapyrrole biosynthesis

We retrieved the genes involved in the tetrapyrrole biosynthesis pathway in Arabidopsis (*A. thaliana*) and maize from the KEGG pathway database (Aoki-Kinoshita and Kanehisa 2007; Kanehisa et al. 2023) We obtained the orthologs of Arabidopsis genes in maize from PLAZA Monocots 5.0 (Van Bel et al. 2022). We retrieved additional genes involved in the tetrapyrrole pathway in maize from the CornCyc database in the Plant Metabolic Network (PMN) data portal (https://pmn.plantcyc.org). The complete list of genes and their correct annotations and nomenclature in maize is provided in Supplementary Table S2.

### Natural variation in transcript abundances

The Box–Cox transformed expression counts were obtained from a previously published study of RNA-sequencing data from 296 diverse maize lines (Kremling et al. 2018). We examined the 70 tetrapyrrole biosynthesis genes expressed in the following 3 leaf tissues: the base of the third leaf (L3Base), mature leaves collected during the day (LMAD), and mature leaves collected during the night (LMAN).

We calculated chlorophyll pathway index values for all 3 leaf tissues using the normalized transformed expression data of the 22 genes encoding the steps in chlorophyll biosynthesis and the chlorophyll cycle (Figs. 1 and 5; Supplementary Table S2). The Z-score for each gene in each tissue of an individual maize line was calculated as follows:


$$Z\text{-}\mathrm{scor}e_{\mathrm{gene}1,\;\mathrm{line}1}=\;\frac{Normalized\;counts\;of\;gene1\;in\;line1\;-Average\;counts\;of\;gene1\;in\;296\;lines}{Standard\;Deviation}$$

Z-scores of 22 chlorophyll pathway genes were averaged for each tissue type to obtain the chlorophyll biosynthesis index for each inbred line.

### Phenotypic data

Phenotypic data were obtained from previously published studies (Khangura et al. 2019, 2022). These included chlorophyll content measurements from the WT and mutant siblings of 343 F_1_ families resulting from crosses between diverse maize lines and *Oy1-N1989/oy1*^B73^. Chlorophyll contents were measured at 2 points using a chlorophyll content meter CCM-200 plus (Opti-Sciences, Inc., Hudson, NH). CCMI represents the first time point of chlorophyll measurement at 25–30 d after sowing, and CCMII is the second chlorophyll measurement from plants at 45–50 d after sowing. The isogenic WT trait values for every mutant in this F1 population provided a perfect case-control design for this study. We used these 2 isogenic genotypes to calculate the ratio of mutant to WT (Ratio CCM) and the difference between WT and mutant (Diff CCM) (Khangura et al. 2022).

### Genome-wide association

SNP data for the association panels were obtained from the imputation of the maize HapMap 3.2.1 data (Bukowski et al. 2018; Khangura et al. 2022). The data were filtered to retain only bi-allelic SNPs. The SNPs were recoded so that all the reference alleles were coded as an “A” and the alternate allele as a “T” and then numericalized to speed computation and simplify the interpretation of allelic effects, as described previously (Khangura et al. 2022). SNPs with a minor allele frequency < 0.05 were removed for each population to reduce false positives. For cis and trans eGWAS and index mapping, data from 296 maize lines were available, and minor allele filtering resulted in 23,913,217 SNPs tested for associations. For the eGWAS analysis, SNPs within 50 kb of each gene's start or stop codon were classified as cis-acting, and the SNPs located at 1Mbp or greater were classified as trans-acting. For the phenotypic data on the F1 families, data from 343 F1 families were available, and minor allele filtering for this population resulted in 22,531,621 SNPs tested for association with each trait. Genome-wide associations were performed by modifying the approach taken in switchgrass (Lovell et al. 2021) to adapt the *bigsnpr* package (Privé et al. 2018) for maize. A preliminary cutoff at a *P*-value threshold ≤ 1 × 10^−4^ was selected to minimize excessive false negative rates. GWAS results were viewed using the interactive browser Zbrowse (Ziegler et al. 2015). Annotations for the candidate genes were obtained from several sources, including Gramene, MaizeGDB (https://www.maizegdb.org) (Portwood et al. 2019; Woodhouse et al. 2021), JGI genome portal (https://genome.jgi.doe.gov/portal/), and Plaza (Proost et al. 2010). Arabidopsis homologs were examined using the Thalemine tool in the Araport project (Krishnakumar et al. 2015, 2016).

### Candidate gene selection for eGWAS trans-acting hotspots

For SNPs affecting the expression of 8 or more genes encoding steps in the tetrapyrrole biosynthesis pathway (Hot SNPs and Hot Top SNPs), up to 3 genes upstream and 3 downstream were tabulated from a 125 kb window around each SNP position (Supplementary Tables S9 and S12). Each gene was annotated with the distance of the gene from the SNP position calculated using the gene's transcription start site. The candidate genes were ranked based on the distance of their transcription start sites to the SNP, with 1 being the closest and 3 being the farthest. A slightly different approach was employed for hotspots due to multiple SNPs within each hotspot. Hotspot boundaries were defined by the minimum and maximum positions of the SNPs within each hotspot. All genes within the hotspot region were retained as potential candidates. In addition, the nearest gene on either side of the hotspot was retained as a candidate if it was within 125 kb of the hotspot edge (Supplementary Table S13).

### Phylogenetic analysis

The protein sequences of the orthogroup containing the maize *les22* gene (Zm00001d029074) were obtained from the Plaza monocot 4.5 databases (Proost et al. 2010). Multiple sequence alignment was performed using clustalW (Larkin et al. 2007) with default parameters (gap opening penalty: 10 and gap extension cost: 0.20). A maximum likelihood tree was estimated using IQ-TREE version 1.5.5 (Nguyen et al. 2015) with substitution model Q.pfam + G4 predicted by Bayesian information criteria (Kalyaanamoorthy et al. 2017). A consensus tree was computed using ultrafast bootstrapping with 1000 replicates (Minh et al. 2013). The tree was visualized using iTOL v6 (Letunic and Bork 2021).

### Accession numbers

Sequence data from this article can be found in the National Library of Medicine's National Center for Biotechnology Information Sequence Read Archive under BioProject number PRJNA1321763. Supplementary Table S2 has the accessions of the pathway genes mentioned in this study.

## Supplementary Material

kiaf431_Supplementary_Data

## Data Availability

The data underlying this article are available in the article and in its online supplementary material.

## Supplementary data

The following materials are available in the online version of this article.

**Supplementary Figure S1.** GO analysis of DEGs in *Oy1-N1989/+* mutants.

**Supplementary Figure S2.** Cis-acting regulatory variation at tetrapyrrole biosynthetic pathway genes.

**Supplementary Figure S3.** Cis-acting polymorphisms at genes encoding steps in tetrapyrrole biosynthetic pathway impact chlorophyll content in *Oy1-N1989/+* mutants.

**Supplementary Figure S4.** SNPs within a 250 kb region around *oy1* associated in trans with transcripts of at least 2 genes encoding steps in tetrapyrrole pathway in LMAD.

**Supplementary Figure S5.** Trans-eQTL hotspots associated with the transcripts of 8 or more genes encoding steps in tetrapyrrole biosynthesis pathway in LMAD within a 40 kb window.

**Supplementary Figure S6.** Trans-eQTL hotspots associated with the transcripts of 8 or more genes encoding steps in tetrapyrrole biosynthesis pathway in LMAN within a 40 kb window.

**Supplementary Figure S7.** Phylogenetic tree for LES22 protein homologs.

**Supplementary Figure S8.** Transcriptional regulators of pathway-level variation in expression of chlorophyll biosynthetic genes.

**Supplementary Table S1.** DEGs in *Oy1-N1989/oy1*^B73^ and *Oy1-N1989/oy1*^Mo17^ compared to their isogenic WT siblings.

**Supplementary Table S2.** Log2 fold change of genes encoding steps in tetrapyrrole biosynthesis pathway in *Oy1-N1989/+* mutants relative to their isogenic WT siblings.

**Supplementary Table S3.** Significant GO terms enriched in DEGs in *Oy1-N1989/oy1*^B73^ and *Oy1-N1989/oy1*^Mo17^ mutants at FDR ≤ 0.05.

**Supplementary Table S4.** Log2 fold change of genes associated with significant GO terms in *Oy1-N1989/oy1*^B73^ and *Oy1-N1989/oy1*^Mo17^ mutants compared to their respective WT siblings.

**Supplementary Table S5.** Cis polymorphisms associated with variation in transcript abundance of genes encoding steps in tetrapyrrole biosynthesis pathway in 3 leaf tissues at *P*-value ≤ 1 × 10^−4^ in GWAS.

**Supplementary Table S6.** Cis SNPs associated with variation in transcript abundance of tetrapyrrole biosynthesis pathway genes that affect chlorophyll content in mutant and WT F1 plants at a *P*-value ≤ 1 × 10^−4^.

**Supplementary Table S7.** SNPs at the *oy1* locus are linked in trans to transcript abundance of genes encoding steps in the tetrapyrrole biosynthesis pathway.

**Supplementary Table S8.** Hot SNPs associated in trans with the transcripts of 8 or more genes encoding steps in tetrapyrrole biosynthesis pathway at GWAS *P* ≤ 1 × 10^−4^ in L3Base, LMAD, and LMAN.

**Supplementary Table S9.** Candidate genes for Hot SNPs affecting the expression of 8 or more tetrapyrrole pathway genes in L3Base, LMAD, and LMAN.

**Supplementary Table S10.** Top SNPs associated in trans with transcript abundance of genes encoding steps in tetrapyrrole biosynthesis pathway in L3Base, LMAD, and LMAN.

**Supplementary Table S11.** Trans-eQTL hotspots affecting the transcripts of 8 or more genes encoding steps in tetrapyrrole biosynthesis pathway within a 40 kb window in L3Base, LMAD, and LMAN.

**Supplementary Table S12.** Candidate genes for hot top SNPs affecting the transcripts of 8 or more tetrapyrrole pathway genes in L3Base, LMAD, and LMAN.

**Supplementary Table S13.** Candidate genes for trans-eQTL hotspots in L3Base, LMAD, and LMAN.

**Supplementary Table S14.** Proportion of trans polymorphisms associated with transcripts of each gene in tetrapyrrole biosynthesis pathway present in trans-eQTL hotspots in 3 leaf tissues.

**Supplementary Table S15.** SNPs within trans-eQTL hotspots that affect chlorophyll content in mutant and WT F1 plants.

**Supplementary Table S16.** SNPs associated with variation in Chlorophyll Biosynthesis Index at *P* ≤ 1 × 10^−4^ in 3 leaf tissues.

**Supplementary Table S17.** Complete DEseq2 output for comparison of *Oy1-N1989/oy1*^B73^ and *Oy1-N1989/oy1*^Mo17^ to their respective WT siblings.

## References

[kiaf431-B1] Albert FW, Kruglyak L. The role of regulatory variation in complex traits and disease. Nat Rev Genet. 2015:16(4):197–212. 10.1038/nrg3891 25707927

[kiaf431-B2] Albus CA, Salinas A, Czarnecki O, Kahlau S, Rothbart M, Thiele W, Lein W, Bock R, Grimm B, Schöttler MA. LCAA, a novel factor required for magnesium protoporphyrin monomethylester cyclase accumulation and feedback control of aminolevulinic acid biosynthesis in tobacco. Plant Physiol. 2012:160(4):1923–1939. 10.1104/pp.112.206045 23085838 PMC3510121

[kiaf431-B3] Anders S, Pyl PT, Huber W. HTSeq–a Python framework to work with high-throughput sequencing data. Bioinformatics. 2015:31(2):166–169. 10.1093/bioinformatics/btu638 25260700 PMC4287950

[kiaf431-B4] Aoki-Kinoshita KF, Kanehisa M. Gene annotation and pathway mapping in KEGG. Methods Mol Biol. 2007:396:71–91. 10.1007/978-1-59745-515-2_6 18025687

[kiaf431-B5] Battersby AR . Tetrapyrroles: the pigments of life. Nat Prod Rep. 2000:17(6):507–526. 10.1039/b002635m 11152419

[kiaf431-B6] Benjamini Y, Yekutieli D. The control of the false discovery rate in multiple testing under dependency. Ann Stat. 2001:29(4):1165–1188. 10.1214/aos/1013699998

[kiaf431-B7] Best NB, Dilkes BP. Transcriptional responses to gibberellin in the maize tassel and control by DELLA domain proteins. Plant J. 2022:112(2):493–517. 10.1111/tpj.15961 36050832 PMC9826531

[kiaf431-B8] Bollivar D, Braumann I, Berendt K, Gough SP, Hansson M. The Ycf54 protein is part of the membrane component of Mg-protoporphyrin IX monomethyl ester cyclase from barley (Hordeum vulgare L.). FEBS J. 2014:281(10):2377–2386. 10.1111/febs.12790 24661504

[kiaf431-B9] Bryant DA, Hunter CN, Warren MJ. Biosynthesis of the modified tetrapyrroles—the pigments of life. J Biol Chem. 2020:295(20):6888–6925. 10.1074/jbc.REV120.006194 32241908 PMC7242693

[kiaf431-B10] Brzezowski P, Richter AS, Grimm B. Regulation and function of tetrapyrrole biosynthesis in plants and algae. Biochim Biophys Acta. 2015:1847(9):968–985. 10.1016/j.bbabio.2015.05.007 25979235

[kiaf431-B11] Bukowski R, Guo X, Lu Y, Zou C, He B, Rong Z, Wang B, Xu D, Yang B, Xie C, et al Construction of the third-generation Zea mays haplotype map. Gigascience. 2018:7(4):1–12. 10.1093/gigascience/gix134 PMC5890452 29300887

[kiaf431-B12] Busch AWU, Montgomery BL. Interdependence of tetrapyrrole metabolism, the generation of oxidative stress and the mitigative oxidative stress response. Redox Biol. 2015:4:260–271. 10.1016/j.redox.2015.01.010 25618582 PMC4315935

[kiaf431-B13] Chen GE, Hunter CN. Protochlorophyllide synthesis by recombinant cyclases from eukaryotic oxygenic phototrophs and the dependence on Ycf54. Biochem J. 2020:477(12):2313–2325. 10.1042/BCJ20200221 32469391 PMC7319587

[kiaf431-B14] Chen Y, Nishimura K, Tokizawa M, Yamamoto YY, Oka Y, Matsushita T, Hanada K, Shirai K, Mano S, Shimizu T. Alternative localization of HEME OXYGENASE 1 in plant cells regulates cytosolic heme catabolism. Plant Physiol. 2024:195(4):2937–2951. 10.1093/plphys/kiae288 38805221

[kiaf431-B15] Cornah JE, Roper JM, Singh DP, Smith AG. Measurement of ferrochelatase activity using a novel assay suggests that plastids are the major site of haem biosynthesis in both photosynthetic and non-photosynthetic cells of pea (Pisum sativum L.). Biochem J. 2002:362(2):423–432. 10.1042/bj3620423 11853551 PMC1222403

[kiaf431-B16] Cornah JE, Terry MJ, Smith AG. Green or red: what stops the traffic in the tetrapyrrole pathway? Trends Plant Sci. 2003:8(5):224–230. 10.1016/S1360-1385(03)00064-5 12758040

[kiaf431-B17] Dailey HA, Dailey TA, Gerdes S, Jahn D, Jahn M, O’Brian MR, Warren MJ. Prokaryotic heme biosynthesis: multiple pathways to a common essential product. Microbiol Mol Biol Rev. 2017:81(1):e00048-16. 10.1128/MMBR.00048-16 28123057 PMC5312243

[kiaf431-B18] Hansson A, Willows RD, Roberts TH, Hansson M. Three semidominant barley mutants with single amino acid substitutions in the smallest magnesium chelatase subunit form defective AAA^+^ hexamers. Proc Natl Acad Sci U S A. 2002:99(21):13944–13949. 10.1073/pnas.212504499 12357035 PMC129802

[kiaf431-B19] Hansson M, Lundqvist J, Sirijovski N, Al-Karadaghi S. Magnesium chelatase: the molecular motor of chlorophyll biosynthesis. In: Ferreira Gloria C, editor. Handbook of porphyrin science: with applications to chemistry, physics, materials Science, engineering, biology and medicine. 28. Singapore: World Scientific; 2013. p. 41–84.

[kiaf431-B20] Herbst J, Girke A, Hajirezaei MR, Hanke G, Grimm B. Potential roles of YCF54 and ferredoxin-NADPH reductase for magnesium protoporphyrin monomethylester cyclase. Plant J. 2018:94(3):485–496. 10.1111/tpj.13869 29443418

[kiaf431-B21] Hu G, Yalpani N, Briggs SP, Johal GS. A porphyrin pathway impairment is responsible for the phenotype of a dominant disease lesion mimic mutant of maize. Plant Cell. 1998:10(7):1095–1105. 10.1105/tpc.10.7.1095 9668130 PMC144048

[kiaf431-B22] Huang M . Characterization and cloning of camouflage1, a maize gene functioning in the tetrapyrrole synthesis pathway [Ph. D. dissertation]. State College (PA): The Pennsylvania State University; 2009.

[kiaf431-B23] Huang M, Slewinski TL, Baker RF, Janick-Buckner D, Buckner B, Johal GS, Braun DM. Camouflage patterning in maize leaves results from a defect in porphobilinogen deaminase. Mol Plant. 2009:2(4):773–789. 10.1093/mp/ssp029 19825655

[kiaf431-B24] Inagaki N, Kinoshita K, Kagawa T, Tanaka A, Ueno O, Shimada H, Takano M. Phytochrome B mediates the regulation of Chlorophyll biosynthesis through transcriptional regulation of ChlH and GUN4 in rice seedlings. PLoS One. 2015:10(8):e0135408. 10.1371/journal.pone.0135408 26270815 PMC4536196

[kiaf431-B25] Jiao Y, Peluso P, Shi J, Liang T, Stitzer MC, Wang B, Campbell MS, Stein JC, Wei X, Chin C-S, et al Improved maize reference genome with single-molecule technologies. Nature. 2017:546(7659):524–527. 10.1038/nature22971 28605751 PMC7052699

[kiaf431-B26] Jurić S, Hazler-Pilepić K, Tomašić A, Lepeduš H, Jeličić B, Puthiyaveetil S, Bionda T, Vojta L, Allen JF, Schleiff E, et al Tethering of ferredoxin:NADP ^+^ oxidoreductase to thylakoid membranes is mediated by novel chloroplast protein TROL. Plant J. 2009:60(5):783–794. 10.1111/j.1365-313X.2009.03999.x 19682289

[kiaf431-B27] Kachroo AH, Laurent JM, Akhmetov A, Szilagyi-Jones M, McWhite CD, Zhao A, Marcotte EM. Systematic bacterialization of yeast genes identifies a near-universally swappable pathway. Elife. 2017:6:e25093. 10.7554/eLife.25093 28661399 PMC5536947

[kiaf431-B28] Kalyaanamoorthy S, Minh BQ, Wong TKF, von Haeseler A, Jermiin LS. ModelFinder: fast model selection for accurate phylogenetic estimates. Nat Methods. 2017:14(6):587–589. 10.1038/nmeth.4285 28481363 PMC5453245

[kiaf431-B29] Kanehisa M, Furumichi M, Sato Y, Kawashima M, Ishiguro-Watanabe M. KEGG for taxonomy-based analysis of pathways and genomes. Nucleic Acids Res. 2023:51(D1):D587–D592. 10.1093/nar/gkac963 36300620 PMC9825424

[kiaf431-B30] Kaur A, Best NB, Hartwig T, Budka J, Khangura RS, McKenzie S, Aragón-Raygoza A, Strable J, Schulz B, Dilkes BP. A maize semi-dwarf mutant reveals a GRAS transcription factor involved in brassinosteroid signaling. Plant Physiol. 2024:195(4):3072–3096. 10.1093/plphys/kiae147 38709680 PMC11288745

[kiaf431-B31] Khangura RS, Johal GS, Dilkes BP. Genome-wide association identifies impacts of chlorophyll levels on reproductive maturity and architecture in maize. bioRxiv 515492. 10.1101/2022.11.07.515492, 08 November 2022, preprint: not peer reviewed.

[kiaf431-B32] Khangura RS, Johal GS, Dilkes BP. Variation in Maize Chlorophyll Biosynthesis alters plant architecture. Plant Physiol. 2020a:184(1):300–315. 10.1104/pp.20.00306 32641472 PMC7479880

[kiaf431-B33] Khangura RS, Marla S, Venkata BP, Heller NJ, Johal GS, Dilkes BP. A *Very Oil Yellow1* modifier of the *Oil Yellow1-N1989* allele uncovers a cryptic phenotypic impact of *Cis* -regulatory variation in Maize. G3 (Bethesda). 2019:9:375–390. 10.1534/g3.118.200798 30518539 PMC6385977

[kiaf431-B34] Khangura RS, Venkata BP, Marla SR, Mickelbart MV, Dhungana S, Braun DM, Dilkes BP, Johal GS. Interaction between induced and natural variation at *oil yellow1* delays reproductive maturity in Maize. G3 (Bethesda). 2020b:10:797–810. 10.1534/g3.119.400838 31822516 PMC7003087

[kiaf431-B35] Kobayashi K, Masuda T. Transcriptional regulation of tetrapyrrole biosynthesis in Arabidopsis thaliana. Front Plant Sci. 2016:7:1811. 10.3389/fpls.2016.01811 27990150 PMC5130987

[kiaf431-B36] Kobayashi K, Masuda T. Transcriptional control for the chlorophyll metabolism. In: Grimm Bernhard, editor. Advances in Botanical Research. 91. San Diego, CA: Academic Press; 2019. p. 133–161.

[kiaf431-B37] Kolde R . pheatmap: pretty heatmaps. R package version 1.0.12; 2019. https://CRAN.R-project.org/package=pheatmap

[kiaf431-B38] Kremling KAG, Chen SY, Su MH, Lepak NK, Romay MC, Swarts KL, Lu F, Lorant A, Bradbury PJ, Buckler ES. Dysregulation of expression correlates with rare-allele burden and fitness loss in maize. Nature. 2018:555:520–523. 10.1038/nature25966 29539638

[kiaf431-B39] Krishnakumar V, Contrino S, Cheng C-Y, Belyaeva I, Ferlanti ES, Miller JR, Vaughn MW, Micklem G, Town CD, Chan AP. ThaleMine: a warehouse for Arabidopsis data integration and discovery. Plant Cell Physiol. 2016:58:e4. 10.1093/pcp/pcw200 28013278

[kiaf431-B40] Krishnakumar V, Hanlon MR, Contrino S, Ferlanti ES, Karamycheva S, Kim M, Rosen BD, Cheng C-Y, Moreira W, Mock SA, et al Araport: the Arabidopsis information portal. Nucleic Acids Res. 2015:43:D1003–D1009. 10.1093/nar/gku1200 25414324 PMC4383980

[kiaf431-B41] Langmead B, Salzberg SL. Fast gapped-read alignment with Bowtie 2. Nat Methods. 2012:9:357–359. 10.1038/nmeth.1923 22388286 PMC3322381

[kiaf431-B42] Larkin MA, Blackshields G, Brown NP, Chenna R, McGettigan PA, McWilliam H, Valentin F, Wallace IM, Wilm A, Lopez R, et al Clustal W and clustal X version 2.0. Bioinformatics. 2007:23:2947–2948. 10.1093/bioinformatics/btm404 17846036

[kiaf431-B43] Larkin RM, Alonso JM, Ecker JR, Chory J. GUN4, a regulator of chlorophyll synthesis and intracellular signaling. Science (1979). 2003:299:902–906. 10.1126/science.1079978 12574634

[kiaf431-B44] Letunic I, Bork P. Interactive Tree Of Life (iTOL) v5: an online tool for phylogenetic tree display and annotation. Nucleic Acids Res. 2021:49:W293–W296. 10.1093/nar/gkab301 33885785 PMC8265157

[kiaf431-B45] Love MI, Huber W, Anders S. Moderated estimation of fold change and dispersion for RNA-seq data with DESeq2. Genome Biol. 2014:15:550. 10.1186/s13059-014-0550-8 25516281 PMC4302049

[kiaf431-B46] Lovell JT, MacQueen AH, Mamidi S, Bonnette J, Jenkins J, Napier JD, Sreedasyam A, Healey A, Session A, Shu S, et al Genomic mechanisms of climate adaptation in polyploid bioenergy switchgrass. Nature. 2021:590(7846):438–444. 10.1038/s41586-020-03127-1 33505029 PMC7886653

[kiaf431-B47] Lundqvist J, Braumann I, Kurowska M, Müller AH, Hansson M. Catalytic turnover triggers exchange of subunits of the magnesium chelatase AAA+ motor unit. J Biol Chem. 2013:288(33):24012–24019. 10.1074/jbc.M113.480012. 23836887 PMC3745346

[kiaf431-B48] Mano NA, Madore B, Mickelbart MV. Different leaf anatomical responses to water deficit in Maize and Soybean. Life. 2023:13:290. 10.3390/life13020290 36836647 PMC9966819

[kiaf431-B49] Minh BQ, Nguyen MAT, von Haeseler A. Ultrafast approximation for phylogenetic Bootstrap. Mol Biol Evol. 2013:30:1188–1195. 10.1093/molbev/mst024 23418397 PMC3670741

[kiaf431-B50] Muszynski MG, Moss-Taylor L, Chudalayandi S, Cahill J, Del Valle-Echevarria AR, Alvarez-Castro I, Petefish A, Sakakibara H, Krivosheev DM, Lomin SN, et al The maize *Hairy Sheath Frayed1* (*Hsf1*) mutation alters leaf patterning through increased cytokinin signaling. Plant Cell. 2020:32:1501–1518. 10.1105/tpc.19.00677 32205456 PMC7203929

[kiaf431-B51] Nguyen L-T, Schmidt HA, von Haeseler A, Minh BQ. IQ-TREE: a fast and effective stochastic algorithm for estimating Maximum-likelihood phylogenies. Mol Biol Evol. 2015:32:268–274. 10.1093/molbev/msu300 25371430 PMC4271533

[kiaf431-B52] Portwood JL, Woodhouse MR, Cannon EK, Gardiner JM, Harper LC, Schaeffer ML, Walsh JR, Sen TZ, Cho KT, Schott DA, et al MaizeGDB 2018: the maize multi-genome genetics and genomics database. Nucleic Acids Res. 2019:47:D1146–D1154. 10.1093/nar/gky1046 30407532 PMC6323944

[kiaf431-B53] Privé F, Aschard H, Ziyatdinov A, Blum MGB. Efficient analysis of large-scale genome-wide data with two R packages: bigstatsr and bigsnpr. Bioinformatics. 2018:34:2781–2787. 10.1093/bioinformatics/bty185 29617937 PMC6084588

[kiaf431-B54] Proost S, Van Bel M, Sterck L, Billiau K, Van Parys T, Van de Peer Y, Vandepoele K. PLAZA: a comparative genomics resource to study gene and genome evolution in plants. Plant Cell. 2010:21:3718–3731. 10.1105/tpc.109.071506 PMC2814516 20040540

[kiaf431-B55] Ryberg M, Terry MJ. Analysis of protochlorophyllide reaccumulation in the phytochrome chromophore-deficient aurea and yg-2 mutants of tomato by in vivo fluorescence spectroscopy. Photosynth Res. 2002:74(2):195–203. 10.1023/A:1020911727791 16228558

[kiaf431-B56] Sawers RJH, Viney J, Farmer PR, Bussey RR, Olsefski G, Anufrikova K, Hunter CN, Brutnell TP. The Maize Oil Yellow1 (Oy1) gene encodes the I subunit of magnesium chelatase. Plant Mol Biol. 2006:60:95–106. 10.1007/s11103-005-2880-0 16463102

[kiaf431-B57] Shi D, Zheng X, Li L, Lin W, Xie W, Yang J, Chen S, Jin W. Chlorophyll deficiency in the maize elongated mesocotyl2 mutant is caused by a defective heme oxygenase and delaying grana stacking. PLoS One. 2013:8:e80107. 10.1371/journal.pone.0080107 24244620 PMC3823864

[kiaf431-B58] Shim K-C, Kang Y, Song J-H, Kim YJ, Kim JK, Kim C, Tai TH, Park I, Ahn S-N. A frameshift mutation in the Mg-chelatase I subunit gene OsCHLI is associated with a lethal chlorophyll-deficient, yellow seedling phenotype in rice. Plants. 2023:12:2831. 10.3390/plants12152831 37570985 PMC10420988

[kiaf431-B59] Stuart D, Sandström M, Youssef HM, Zakhrabekova S, Jensen PE, Bollivar DW, Hansson M. Aerobic barley Mg-protoporphyrin IX monomethyl ester cyclase is powered by electrons from ferredoxin. Plants. 2020:9:1157. 10.3390/plants9091157 32911631 PMC7570240

[kiaf431-B60] Tanaka R, Kobayashi K, Masuda T. Tetrapyrrole metabolism in *Arabidopsis thaliana*. Arabidopsis Book. 2011:9:e0145. 10.1199/tab.0145 22303270 PMC3268503

[kiaf431-B61] Tanaka R, Tanaka A. Tetrapyrrole biosynthesis in higher plants. Annu Rev Plant Biol. 2007:58:321–346. 10.1146/annurev.arplant.57.032905.105448 17227226

[kiaf431-B62] Tian T, Liu Y, Yan H, You Q, Yi X, Du Z, Xu W, Su Z. agriGO v2.0: a GO analysis toolkit for the agricultural community, 2017 update. Nucleic Acids Res. 2017:45:W122–W129. 10.1093/nar/gkx382 28472432 PMC5793732

[kiaf431-B63] Van Bel M, Silvestri F, Weitz EM, Kreft L, Botzki A, Coppens F, Vandepoele K. PLAZA 5.0: extending the scope and power of comparative and functional genomics in plants. Nucleic Acids Res. 2022:50:D1468–D1474. 10.1093/nar/gkab1024 34747486 PMC8728282

[kiaf431-B64] Vavilin DV, Vermaas WFJ. Regulation of the tetrapyrrole biosynthetic pathway leading to heme and chlorophyll in plants and cyanobacteria. Physiol Plant. 2002:115:9–24. 10.1034/j.1399-3054.2002.1150102.x 12010463

[kiaf431-B65] Walker JC, Willows DR. Mechanism and regulation of Mg-chelatase. Biochem J. 1997:327:321–333. 10.1042/bj3270321 9359397 PMC1218797

[kiaf431-B66] Walter W, Sánchez-Cabo F, Ricote M. GOplot: an R package for visually combining expression data with functional analysis. Bioinformatics. 2015:31:2912–2914. 10.1093/bioinformatics/btv300 25964631

[kiaf431-B67] Wang P, Ji S, Grimm B. Post-translational regulation of metabolic checkpoints in plant tetrapyrrole biosynthesis. J Exp Bot. 2022:73:4624–4636. 10.1093/jxb/erac203 35536687 PMC9992760

[kiaf431-B68] Willows RD, Hansson M. Mechanism, structure, and regulation of magnesium chelatase. In: Kadish K, Smith K, Guilard R, editors. The porphyrin handbook. 13. San Diego (CA): Academic Press; 2003. p. 1–47.

[kiaf431-B69] Woodhouse MR, Cannon EK, Portwood JL, Harper LC, Gardiner JM, Schaeffer ML, Andorf CM. A pan-genomic approach to genome databases using maize as a model system. BMC Plant Biol. 2021:21:385. 10.1186/s12870-021-03173-5 34416864 PMC8377966

[kiaf431-B70] Xue Y, Dong H, Huang H, Li S, Shan X, Li H, Liu H, Xia D, Su S, Yuan Y. Mutation in Mg-protoporphyrin IX monomethyl ester (oxidative) cyclase gene ZmCRD1 causes chlorophyll-deficiency in maize. Front Plant Sci. 2022:13:912215. 10.3389/fpls.2022.912215 35873969 PMC9301084

[kiaf431-B71] Zhao Y, Xu W, Wang L, Han S, Zhang Y, Liu Q, Liu B, Zhao X. A maize necrotic leaf mutant caused by defect of coproporphyrinogen III oxidase in the porphyrin pathway. Genes (Basel). 2022:13:272. 10.3390/genes13020272 35205317 PMC8872553

[kiaf431-B72] Ziegler GR, Hartsock RH, Baxter I. Zbrowse: an interactive GWAS results browser. PeerJ Comput Sci. 2015:1:e3. 10.7717/peerj-cs.3

