# Supplementary material for: Natural variation in tetrapyrrole biosynthetic enzymes and their regulation modifies the maize chlorophyll mutant Oy1-N1989
Source: Plant Physiol. 2025 Nov 12;199(3):kiaf431. doi: 10.1093/plphys/kiaf431 (PMC12607265; doi:10.1093/plphys/kiaf431)
Supplement: kiaf431_Supplementary_Data [file kiaf431_supplementary_data.zip › Supplemental text.docx]

**Supplemental text**

GO term analysis

To complement the DEG analysis, we also looked at gene ontology term enrichment in the DEG from the *Oy1-N1989* mutants in phenotypically mild and severe genetic backgrounds. DEGs in phenotypically mild *Oy1-N1989/oy1*^B73^ were significantly enriched in GO terms associated with six biological processes, including photosynthesis (GO:0015979), lipid metabolic process (GO:0006629), response to abiotic stimulus (GO:0009628) and embryo development (GO:0009790) (Supplemental Figure S1). Among the cellular components, the most enriched GO terms were plastid (GO:0009536) and thylakoid (GO:0009579) (Supplemental Figure S1). A greater proportion of transcripts in the GO terms cited above were increased in the mutant in less phenotypically impacted genetic background, *Oy1-N1989/oy1*^B73^ (Supplemental Figure S1)*.* DEGs in severe *Oy1-N1989/oy1*^Mo17^ were enriched in fifty-three significant GO terms, including 34 for biological processes and 19 for cellular components (Supplemental Table S3). Similar to *Oy1-N1989/oy1*^B73^*,* DEGs in *Oy1-N1989/oy1*^Mo17^ were enriched in photosynthesis (GO:0015979), response to abiotic stimulus (GO:0009628), lipid metabolic process (GO:0006629) and embryo development (GO:0009790) (Supplemental Figure S1, Supplemental Table S3). However, as depicted by the z-score values, more genes associated with these terms were down-regulated in *Oy1-N1989/oy1*^Mo17^, suggesting that enhancement of the mutant resulted in an opposite impact for some processes described by GO terms. This might be due to a biological change in the regulation of a true process or an artifact of the shift in the overlapping sets of genes within a GO term and our two DEG sets. A detailed examination of the genes within each term in demonstrated that the DEGs affected in each of the mutant backgrounds were impacted in the same direction even for the significant GO terms with opposite expression directions between the two experiments (Supplemental Table S4). The overall opposite effects of the enhancing and suppressing backgrounds on the mutant DEG sets overlapping these GO term sets resulted from the inclusion of additional DEG in the severe background DEG set*.* Given the overwhelming concordance of expression directions at individual genes for the *Oy1-N1989*/+ mutants in these two backgrounds (Figures 2 and 3), this highlights a pitfall in interpreting gene expression changes via GO terms. In addition to the above-mentioned biological processes, *Oy1-N1989/oy1*^Mo17^ DEGs were enriched in carbohydrate metabolic process (GO:0005975), reproduction (GO:0000003, GO:0022414), anatomical structure development (GO:0048856, GO:0048608), regulation of cell size (GO:0008361), cell growth (GO:0016049), cellular homeostasis (GO:0019725) and cell communication (GO:0007154). Genes associated with these processes were predominantly down-regulated in *Oy1-N1989/oy1*^Mo17^. Among the cellular components, plastid (GO:0009536) and thylakoid (GO:0009579) were the most enriched GO terms in *Oy1-N1989/oy1*^Mo17^ DEGs (Supplemental Figure S1).
