# Supplementary material for: Natural variation in tetrapyrrole biosynthetic enzymes and their regulation modifies the maize chlorophyll mutant Oy1-N1989
Source: Plant Physiol. 2025 Nov 12;199(3):kiaf431. doi: 10.1093/plphys/kiaf431 (PMC12607265; doi:10.1093/plphys/kiaf431)
Supplement: kiaf431_Supplementary_Data [file kiaf431_supplementary_data.zip › Supplementary Data.pdf]

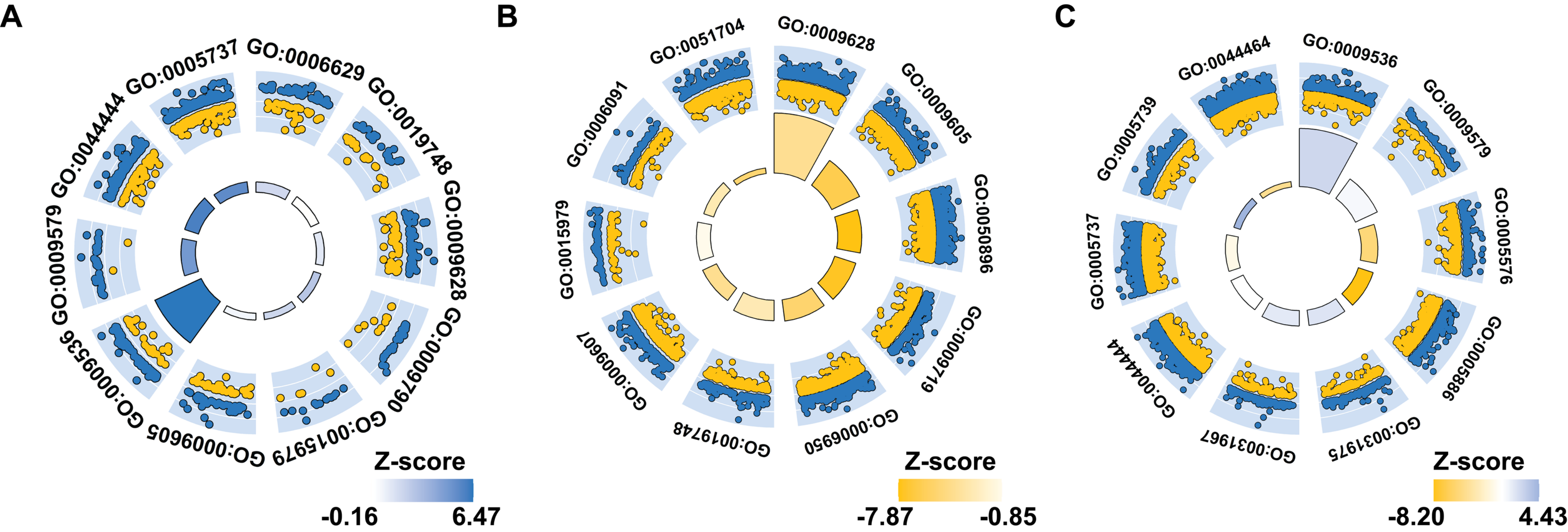

GO ID	Term	Category	No. of Genes
GO:0006629	lipid metabolic process	BP	80
GO:0019748	secondary metabolic process	BP	41
GO:0009628	response to abiotic stimulus	BP	99
GO:0009790	embryo development	BP	38
GO:0015979	photosynthesis	BP	18
GO:0009605	response to external stimulus	BP	77
GO:0009536	plastid	CC	138
GO:0009579	thylakoid	CC	31
GO:0044444	cytoplasmic part	CC	251
GO:0005737	cytoplasm	CC	284

GO ID	Term	Category	No. of Genes
GO:0009628	response to abiotic stimulus	BP	728
GO:0009605	response to external stimulus	BP	531
GO:0050896	response to stimulus	BP	2081
GO:0009719	response to endogenous stimulus	BP	530
GO:0006950	response to stress	BP	1201
GO:0019748	secondary metabolic process	BP	249
GO:0009607	response to biotic stimulus	BP	391
GO:0015979	photosynthesis	BP	111
GO:0006091	generation of precursor metabolites and energy	BP	185
GO:0051704	multi-organism process	BP	517
GO:0005975	carbohydrate metabolic process	BP	441
GO:0006629	lipid metabolic process	BP	419

GO ID	Term	Category	No. of Genes
GO:0009536	plastid	CC	711
GO:0009579	thylakoid	CC	163
GO:0005576	extracellular region	CC	321
GO:0005886	plasma membrane	CC	694
GO:0031975	envelope	CC	271
GO:0031967	organelle envelope	CC	266
GO:0044444	cytoplasmic part	CC	1504
GO:0005737	cytoplasm	CC	1748
GO:0005739	mitochondrion	CC	360
GO:0044464	cell part	CC	2552

log2FC ● *Oy1-N1989/+* Induced ● *Oy1-N1989/+* Repressed

Figure S1. Gene Ontology (GO) analysis of differentially expressed genes (DEGs) in *Oy1-N1989/+* mutants. Circle plots show top significant GO terms for biological processes (BP) and cell component (CC) enriched in DEGs in A) *Oy1-N1989/oy1*^{B73} and (B, C) *Oy1-N1989/oy1*^{Mo17} mutants at FDR≤ 0.05. The outer circle depicts the log2 fold change of DEGs in *Oy1-N1989/+* mutants as compared to their wild-type (WT) siblings for each enriched GO term. Blue dots indicate genes induced in *Oy1-N1989/+* as compared to WT and gold dots indicate genes repressed in *Oy1-N1989/+*. The color of the inner circle represents the Z-score, and the thickness represents significance of GO term (-log10 (p-value)).

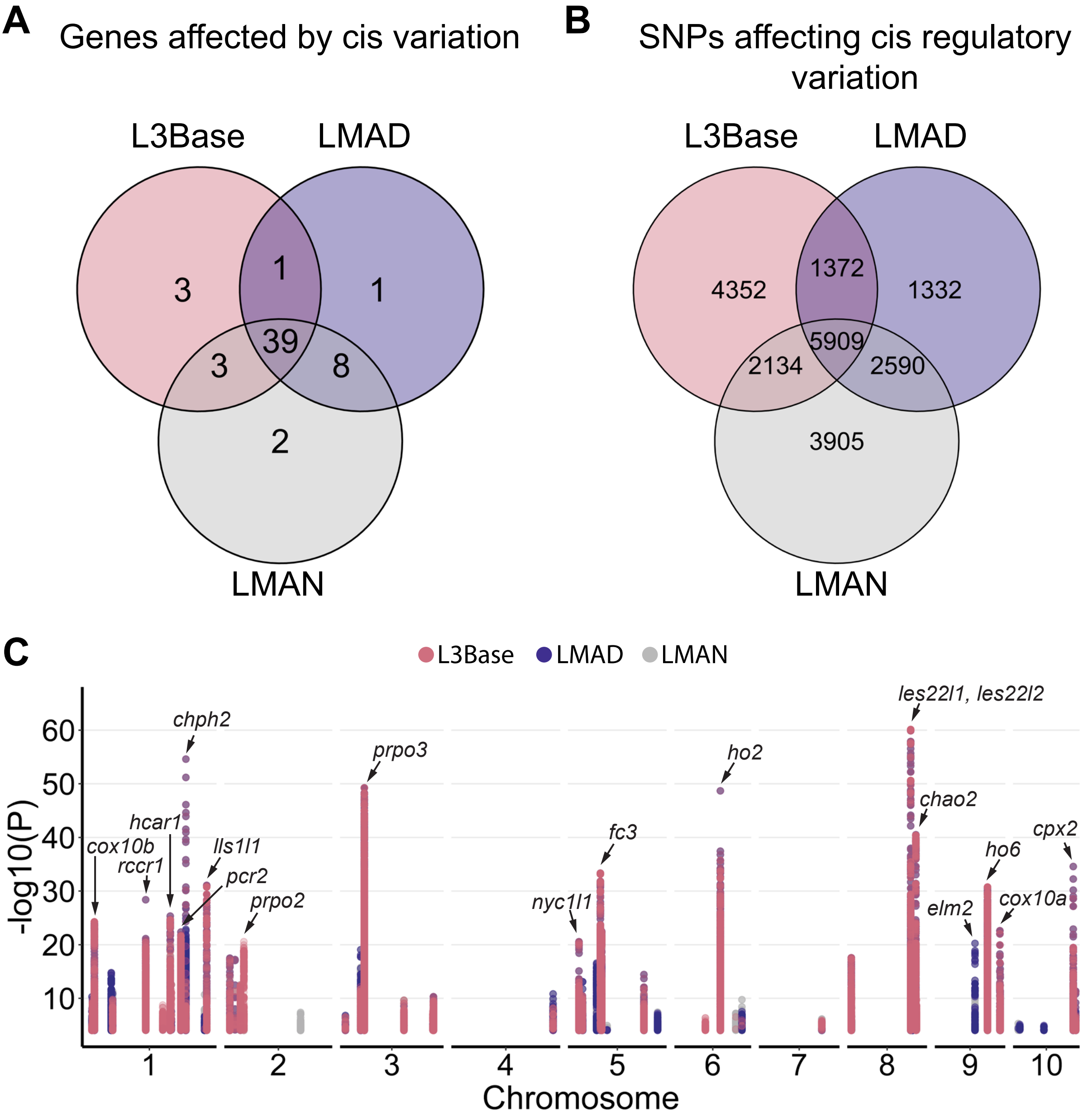

Figure S2. Cis-acting regulatory variation at tetrapyrrole biosynthetic pathway genes. A) Distribution of number of genes in tetrapyrrole biosynthetic pathway whose expression was affected by cis polymorphisms in three leaf tissues; B) Distribution of cis-acting SNPs affecting expression of tetrapyrrole biosynthetic genes detected in three leaf tissues.; C) Manhattan plot showing cis-acting SNPs associated with expression of tetrapyrrole biosynthetic pathway genes at p-value < $1x10^{-4}$. The genes that were associated with SNPs at p-value <$1x10^{-20}$ are labelled. The colors indicate cis polymorphisms detected in three leaf tissues: L3Base (pink), LMAD (purple) and LMAN (grey).

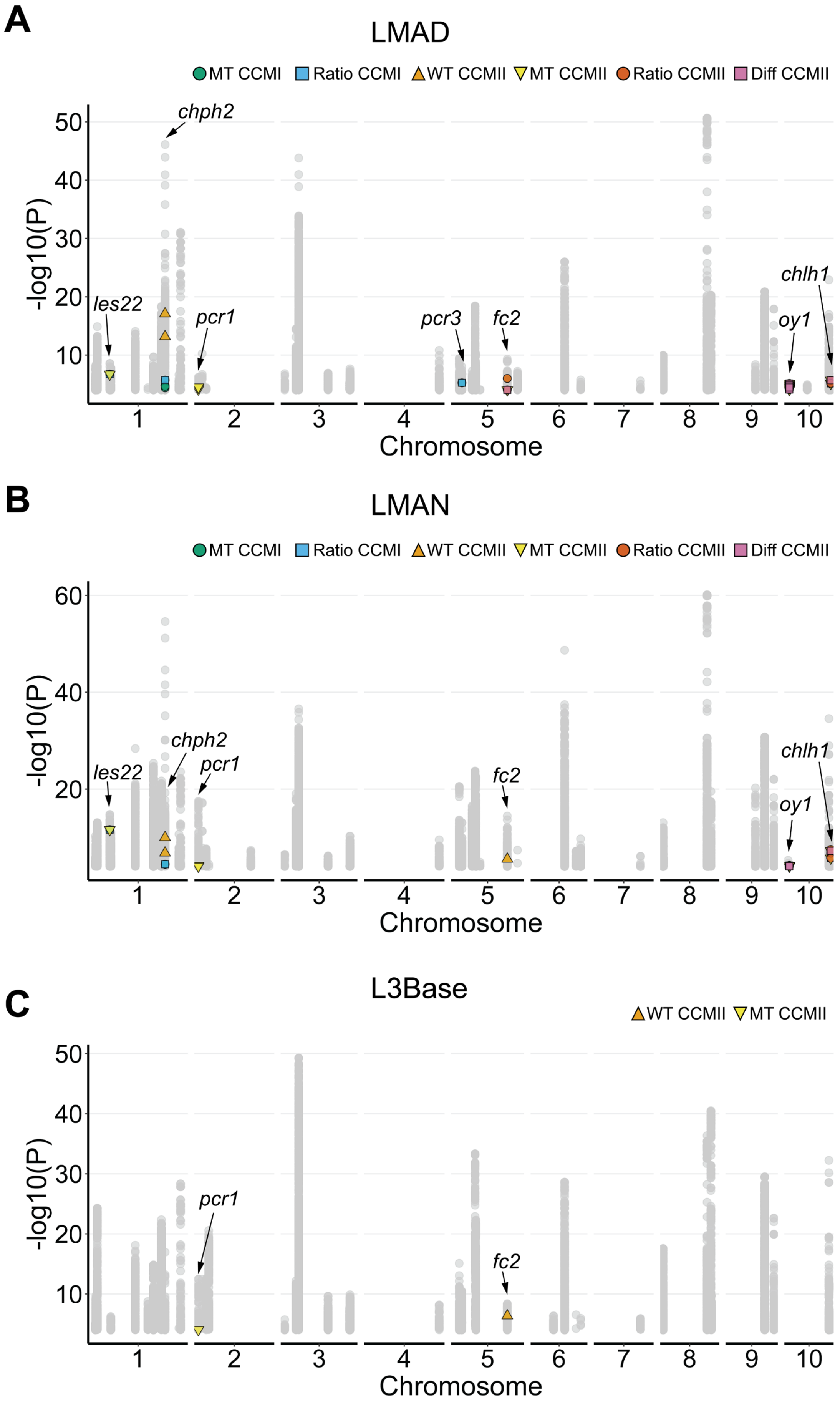

Figure S3. Cis-acting polymorphisms at genes encoding steps in tetrapyrrole biosynthetic pathway impact chlorophyll content in *Oy1-N1989/+* mutants. Grey dots depict the cis-polymorphisms associated with expression of tetrapyrrole pathway genes in A) LMAD, B) LMAN and C) L3Base identified through eGWAS at p-value < $1x10^{-4}$ The colored shapes highlight the SNPs that had significant effect on chlorophyll accumulation traits at p-value ≤ $1x10^{-4}$.

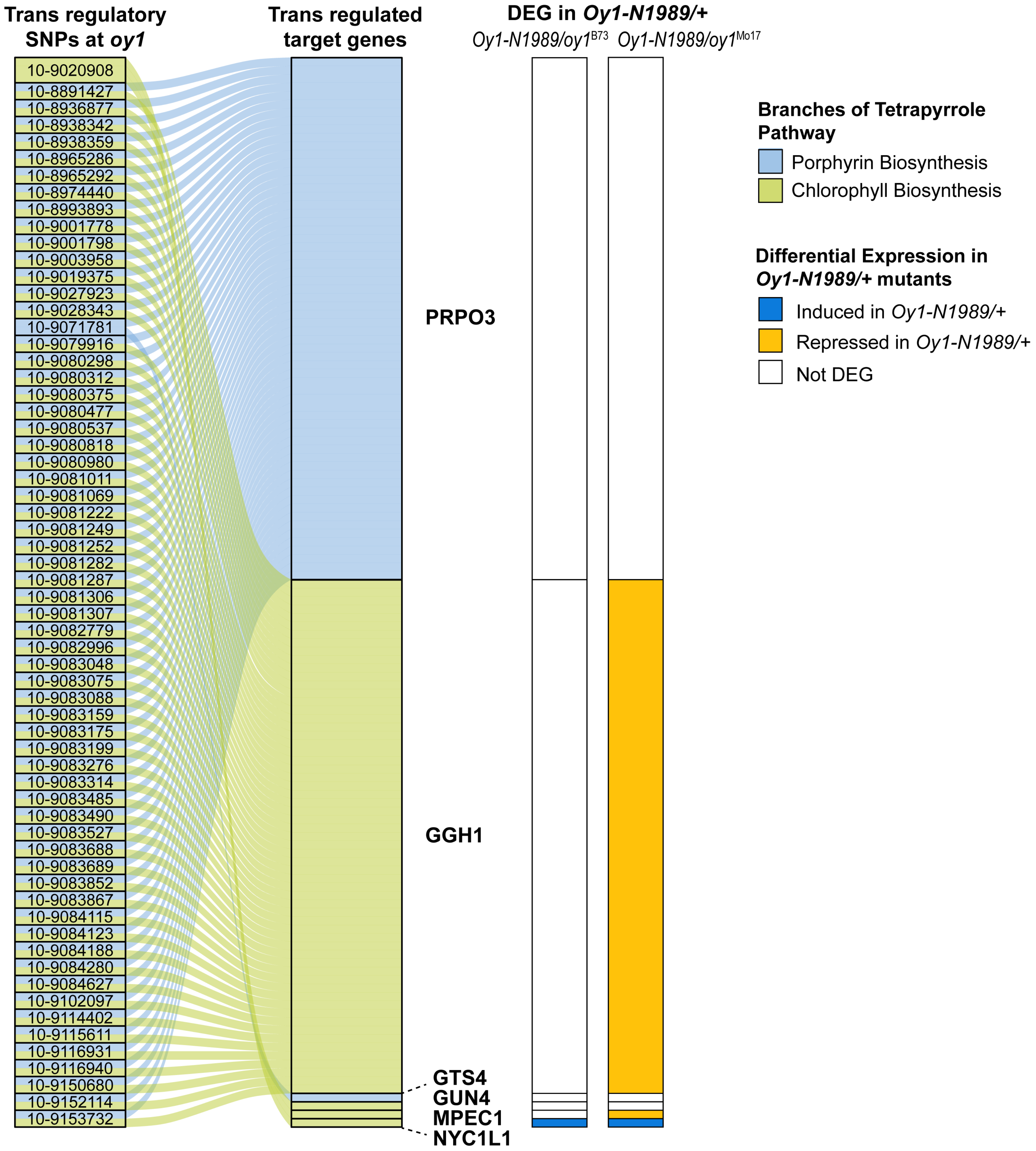

Figure S4. SNPs within a 250kb region around *oy1* associated in trans with transcripts of at least two genes encoding steps in tetrapyrrole pathway in LMAD. Light blue and green colors indicate the branches of the tetrapyrrole pathway. The panels on the right depict the direction of effect of *Oy1-N1989* on transcript accumulation in mild and severe *Oy1-N1989/+* mutants as compared to their respective congenic wild types if they exceeded an unadjusted p-value ≤ 0.05 in RNA-seq analysis. Dark blue color indicates genes induced in the mutant and gold color indicates genes repressed in the mutant. White indicates that the genes were not differentially expressed at p-value ≤ 0.05.

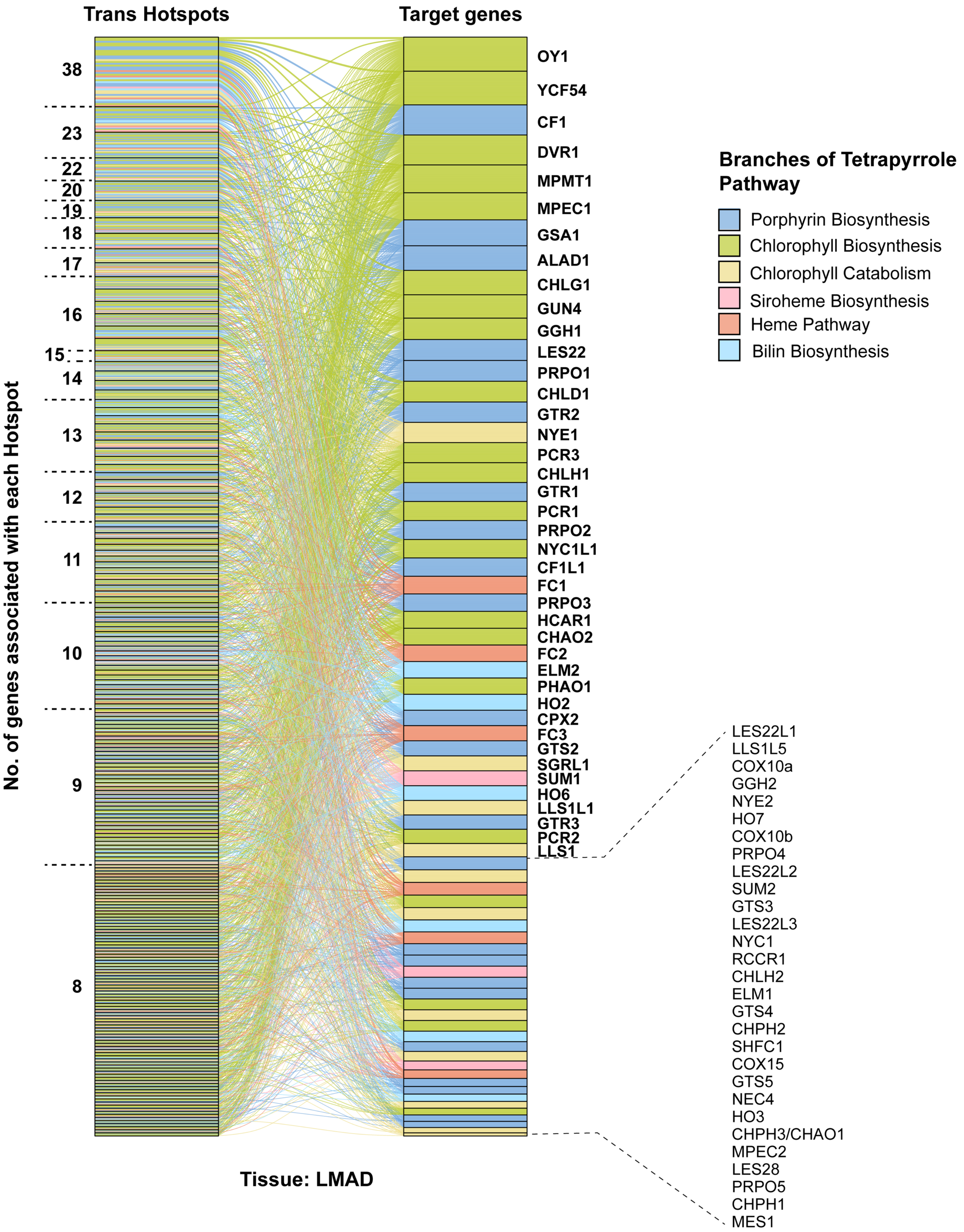

Figure S5. Trans eQTL hotspots associated with the transcripts of eight or more genes encoding steps in tetrapyrrole biosynthesis pathway in LMAD within a 40 kb window. The colors indicate different branches of tetrapyrrole biosynthetic pathway.

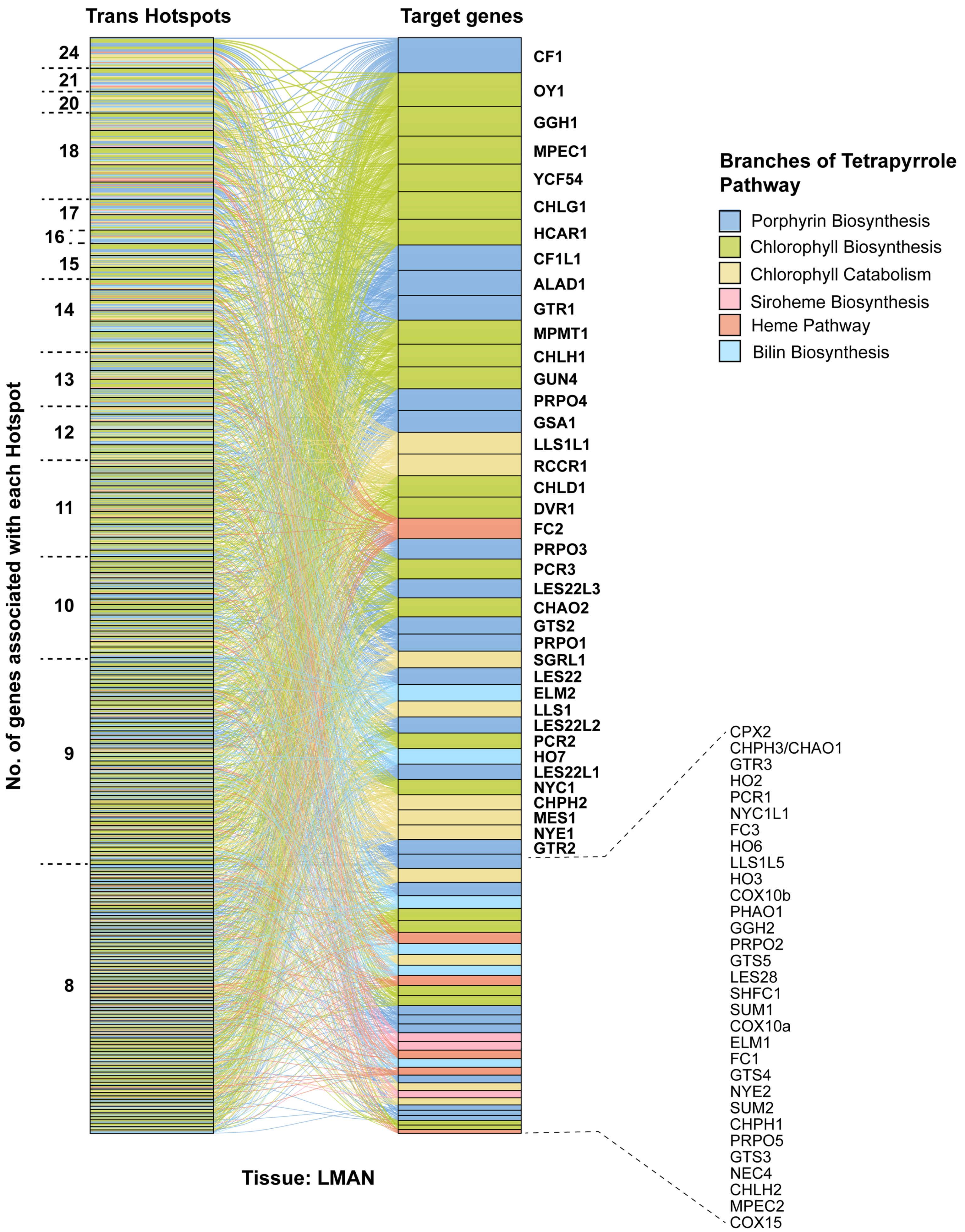

Figure S6. Trans eQTL hotspots associated with the transcripts of eight or more genes encoding steps in tetrapyrrole biosynthesis pathway in LMAN within a 40 kb window. The colors indicate different branches of tetrapyrrole biosynthetic pathway.

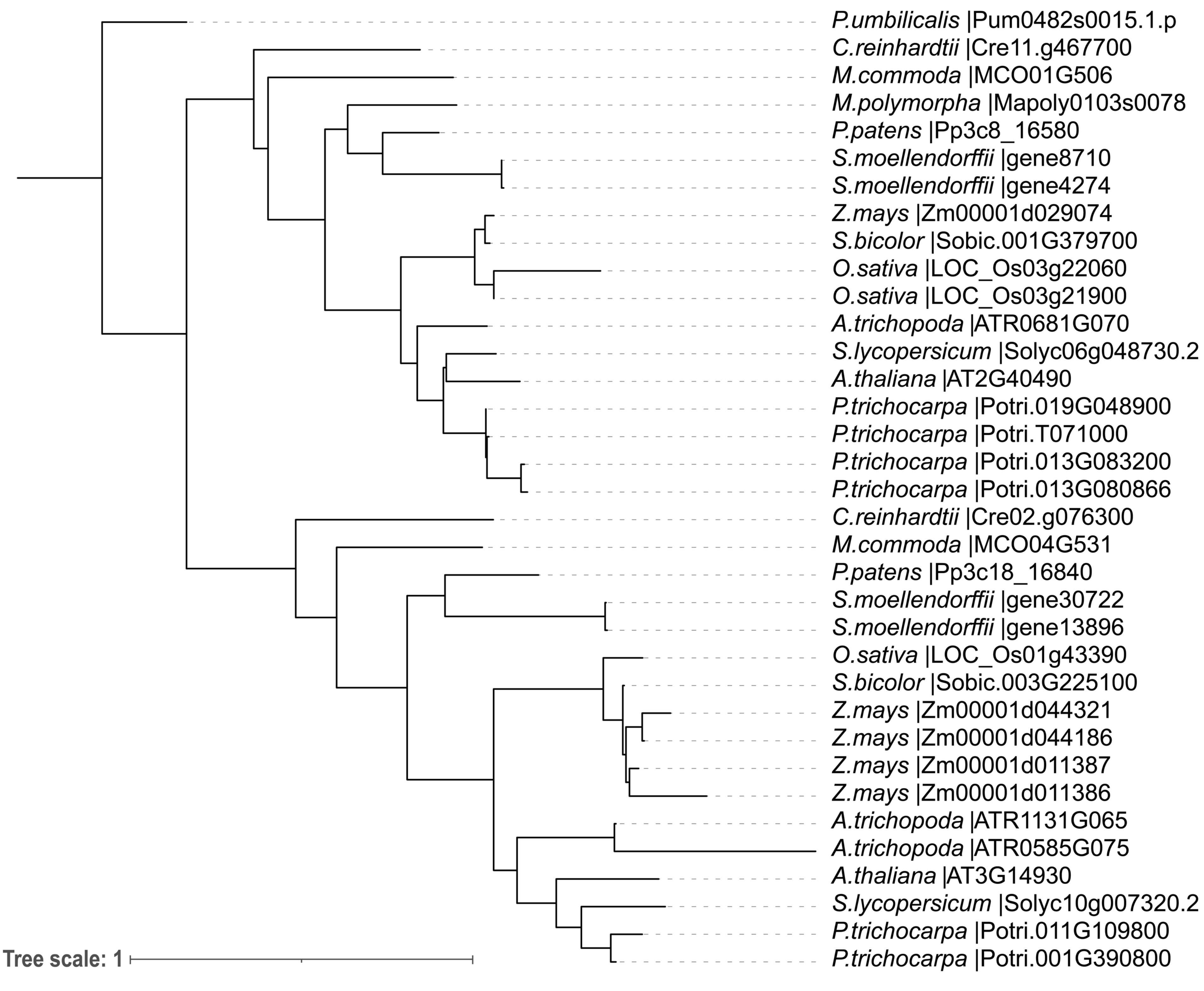

Figure S7. Phylogenetic tree for LES22 protein homologs.

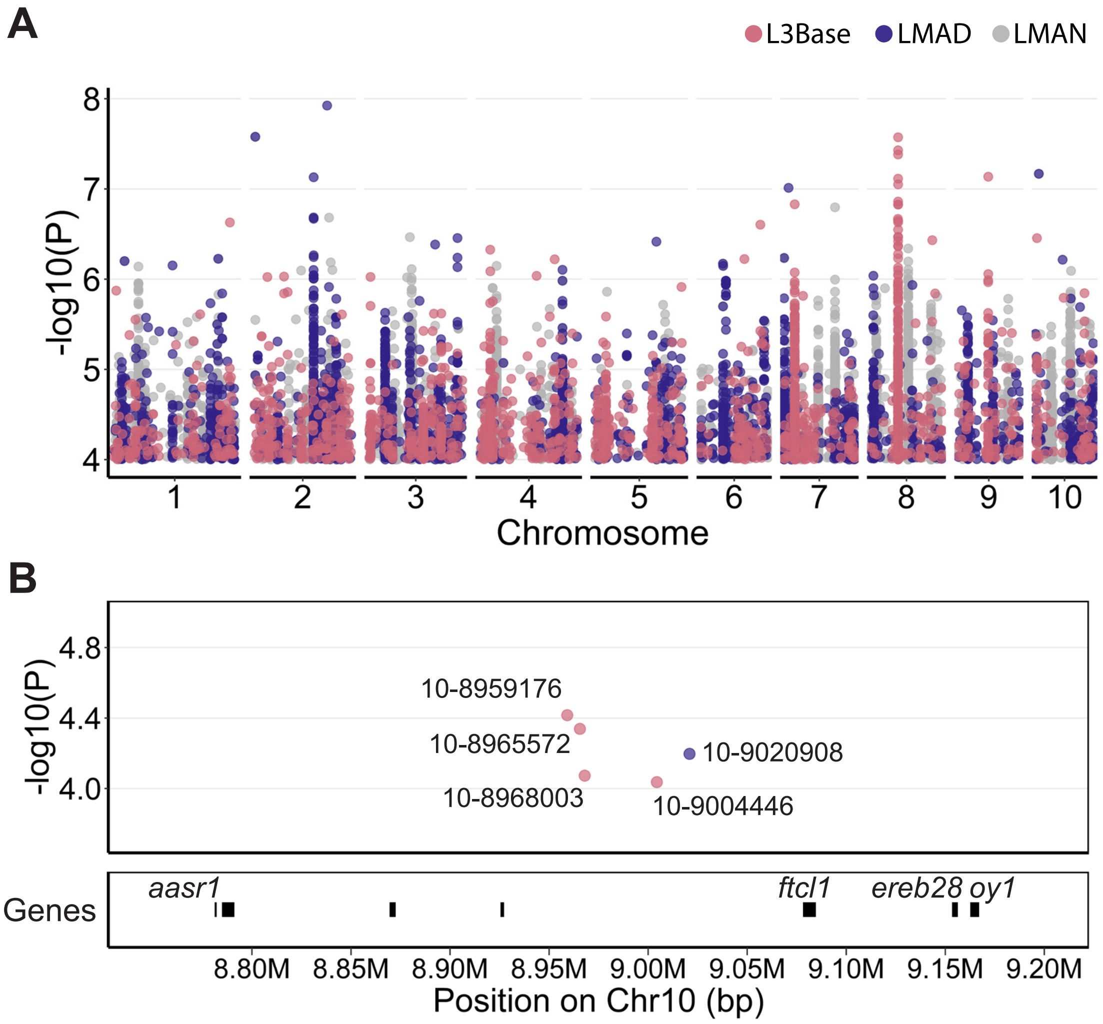

Figure S8. Transcriptional regulators of pathway level variation in expression of chlorophyll biosynthetic genes. A) Manhattan plot showing the SNPs associated with variation in chlorophyll biosynthesis index (calculated from 22 chlorophyll pathway genes) in three leaf tissues: L3Base (pink), LMAD (purple) and LMAN (grey) at GWAS p-value < $1x10^{-4}$; B) SNPs at *oy1* locus that affect chlorophyll biosynthesis index.
